# Pericapsular nerve group block reduces opioid use and pain after hip surgery: A systematic review and meta-analysis of randomized controlled trials

**DOI:** 10.1371/journal.pone.0310008

**Published:** 2024-11-08

**Authors:** Xianghong Hu, Dahao Chenyang, Bin Xu, Yangjun Lao, Hongfeng Sheng, Shuliang Zhang, Yuliang Huang

**Affiliations:** 1 Orthopedics, Tongde Hospital of Zhejiang Province, Hangzhou, China; 2 Orthopedics, The Second Affiliated Hospital of Zhejiang Chinese Medical University, Hangzhou, China; Stanford University School of Medicine, UNITED STATES OF AMERICA

## Abstract

**Background:**

While the pericapsular nerve group (PENG) block has become increasingly popular for managing pain after hip surgery, its efficacy remains controversial.

**Methods:**

We systematically searched Pubmed, Web of Science, Embase, and the Cochrane Library for randomized controlled trials to assess current evidence about the efficacy of the PENG block. Patients who received PENG block were compared to those who received sham/no block in terms of opioid consumption and pain within 24h after surgery, time to first opioid requirement, functional recovery, risk of nausea and vomiting, and patient dissatisfaction. The quality of evidence was assessed using the "Grading of Recommendations Assessment, Development and Evaluation" (GRADE) system.

**Results:**

We meta-analyzed six trials involving 416 patients who received preoperative PENG block and 415 who received sham/no block. Patients did not receive any other type of multimodal analgesia. Within 24 h after hip surgery, PENG block significantly reduced postoperative opioid consumption (MD = -12.03, 95% CI: -21.47 to -2.59, *P* < 0.01, *I*^*2*^ = 97%), particularly in subpopulations undergoing hip replacement, hip fracture, or who had spinal anesthesia, and it significantly decreased dynamic pain scores, but not static scores, without increasing risk of nausea or vomiting or patient dissatisfaction. Individual studies suggested that the PENG block can prolong the time to the first opioid requirement and can improve functional recovery. Most meta-analyses provided evidence of moderate quality according to the GRADE system.

**Conclusions:**

The available evidence indicates that preoperative PENG block can significantly decrease opioid consumption and pain early after hip surgery, and it may also promote early functional rehabilitation. However, the limited number of included studies and sample size make it difficult to draw firm conclusions. The decision on whether to apply the PENG block should take into account the patient’s age and the type of surgery and anesthesia.

## 1. Introduction

Hip fractures are a significant public health concern globally and are expected to occur in 6.3 million people annually by 2050 as populations age [1]. They have sometimes been called the "last fracture of life" because they are associated with numerous complications as well as rates of disability and mortality [2–4], which place a significant healthcare burden on society and the economy [5–7].

Most patients experience pain after hip surgery, leading them to limit their activities [8], which can compromise functional recovery [9] and can shorten years of healthy living [10]. One way to reduce such pain is by giving patients opioids perioperatively, which are effective analgesics but increase the risk of nausea and vomiting [11], respiratory depression [12], cognitive impairment [13], and even mortality [14]. The application of multimodal analgesia has been shown to decrease the amounts of opioids required for pain management [15]. In recent years, a pericapsular nerve group (PENG) block has emerged as a promising approach for selectively blocking the articular branches of the femoral, obturator, and accessory obturator nerves, while preserving motor function [16].

Some randomized controlled trials (RCTs) have demonstrated the effectiveness of the PENG block in diminishing opioid consumption, an important outcome in pain management, while also maintaining the functionality of the quadriceps muscles, critical for patient mobility [17, 18]. However, other research has indicated no significant difference in analgesic efficacy or opioid consumption when comparing the PENG block to placebo interventions [19, 20]. One problem is that studies have varied in whether they compared patients who received PENG block to patients who received fascia iliaca compartment block, femoral nerve block, or peri-articular injection of local anesthetic [21–23].

To clarify the current evidence on the efficacy of the PENG block, we meta-analyzed only RCTs that compared patients who received this block to patients who received sham/no block without any other type of multimodal analgesia.

## 2. Methods

This study was conducted and reported according to the Preferred Reporting Items for Systematic Reviews and Meta-Analyses (PRISMA) statement (S1 Appendix) and registered on PROSPERO (CRD42023483095).

### 2.1 Search strategy

Pubmed, Embase, Web of Science, and Cochrane Library were searched through August 30, 2023, using combinations of the terms “Hip surgery”, “pericapsular nerve group or PENG”, and “placebo or no block” (S2 Appendix). The reference lists of review articles were also searched manually. No language restrictions were imposed during study selection. Two investigators (XHH and DHCY) conducted their searches independently, and then discrepancies were resolved by a third investigator (YLH).

### 2.2 Inclusion criteria

To be included, studies had to have a randomized controlled design involving patients at least 18 years old who underwent hip surgery, during which they received a PENG block or sham/no block for analgesia. Studies had to report one or more of the following outcomes: pain scores, opioid consumption, nausea and vomiting, patient dissatisfaction, and functional recovery. Studies were excluded if patients received combinations of PENG block with other interventions.

### 2.3 Data extraction

Two investigators (XHH and DHCY) independently extracted on 23 August 2023 the following information from included studies using a predefined form: investigator, year, country, patient demographics, type of surgery, anesthetic and dose, block timing (pre- or peri-operative), and outcomes. Intravenous morphine or fentanyl equivalents were converted to oral morphine equivalents as described [24]. If studies reported outcomes in the "post-anesthesia care unit", we defined that as within 1 h after surgery. For missing data, the corresponding author was contacted by e-mail. If there is a disagreement, another author (YLH) will assist in resolving it.

### 2.4 Outcomes evaluation

The primary outcomes of this study were to compare opioid consumption, and static and dynamic pain scores between the PENG group and the sham/no block group over the 24 h post-operatively. Secondary outcomes were time to the first opioid requirement, functional recovery, risk of nausea and vomiting events, and patient satisfaction.

### 2.5 Quality of evidence

The risk of bias in included studies was assessed using the Cochrane tool "Risk of Bias 2". The quality of evidence from our meta-analyses was assessed using the "Grading of recommendations assessment, development and evaluation" (GRADE) system [25]. Then discrepancies were resolved by a third investigator.

### 2.6 Statistical analysis

Data were analyzed statistically using R 4.3.1, and meta-analyses were performed using the *meta* package in R. Continuous variables were meta-analyzed in terms of the mean difference (MD) using the "Metacont" function, while binary variables were meta-analyzed in terms of the risk ratio (RR) using the "Metabin" function. Effect sizes were reported together with a 95% confidence interval (95%CI). All meta-analyses involved a random-effects model because of the substantial variation across studies. Results that could not be analyzed quantitatively were described qualitatively (HFS and SLZ). All data extracted for quantitative analysis are presented in S3 Appendix. Discrepancies were resolved by discussion with a third researcher (YLH).

Heterogeneity in a meta-analysis was considered significant if *I*^*2*^ > 50%. We assessed the robustness of meta-analyses by repeating them after deleting each study one at a time. We did not assess the included studies for risk of publication bias because there were fewer than 10 [26].

## 3. Results

### 3.1 Search results

**Fig 1:**
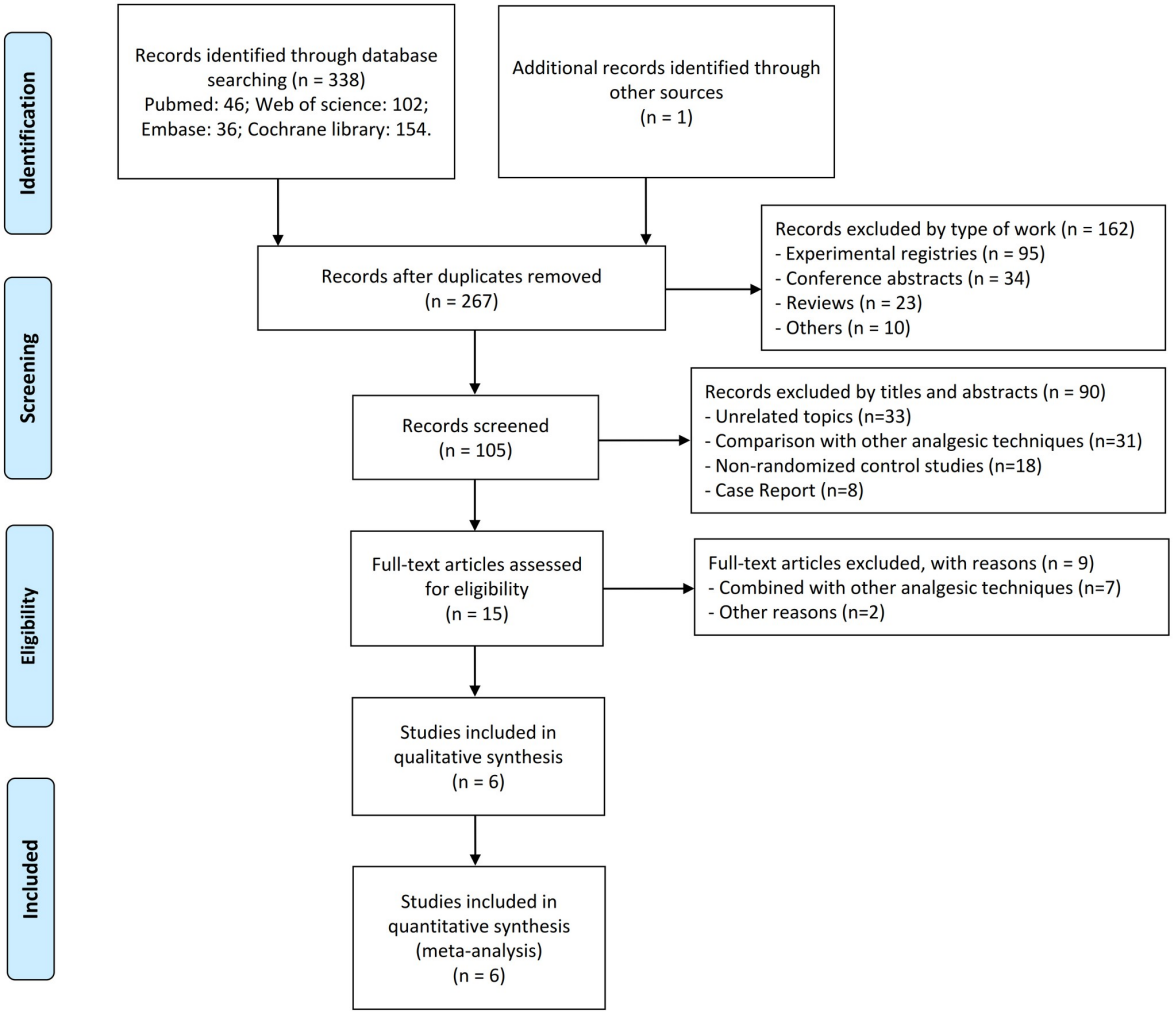
PRISMAS flowchart of the screening process.

We initially retrieved 339 potentially relevant publications from four electronic databases and supplementary resources. After the meticulous removal of duplicates, this number was reduced to 267. Following a stringent screening process that eliminated experimental registries, conference abstracts, review articles, and non-peer-reviewed documents such as letters and errata,105 publications were left for further evaluation. Upon reviewing the titles and abstracts, 90 publications were excluded, and after a thorough full-text review, an additional nine were deemed ineligible for inclusion (S4 Appendix). Fig 1 illustrates the literature screening process. In the end, six RCTs were included in the systematic review and meta-analysis (Table 1), involving 416 patients who received PENG block and 415 who received either sham PENG block or, in one study [19], no block at all. Two trials were conducted in the United States [19, 20], and one each in South Korea [27], Poland [17], Germany [28], and Singapore [29]. Types of surgery were total hip arthroplasty [17, 19], hip arthroscopy [20, 28], and hip fracture surgery [27, 29]. The two studies involving hip arthroscopy involved patients no older than 58 years. The type of anesthesia was general [20, 27, 28] or spinal [17, 19, 29]. Two studies were published in 2022 and four in 2023.

**Table 1:** Characteristics of the randomized controlled trials in this review.

Study	Country	Type of surgery	Anesthesia	Block timing	Comparison	Age (yr), PENG/Control	No. patients, PENG/Control	No. male, PENG/Control	Type of block		Outcomes reported
									PENG	Control	
Amato et al., 2022 [20]	USA	Hip arthroscopy	General	Preoperative	PENG vs Sham	32.5±10.2 / 29.4±9.9	34/34	15/14	20 mL 0.5% ropivacaine	5 mL 0.9% saline	①②③④
Chung et al., 2022 [27]	South Korea	Hip fracture surgery	General	Preoperative	PENG vs Sham	75.96±7.54 / 72.60±9.66	25/25	11/12	25 mL 0.5% ropivacaine	25 mL saline	①②③④⑤⑥
Domagalska et al., 2023 [17]	Poland	Total hip arthroplasty	Spinal	Preoperative	PENG vs Sham	66.0±5.8 / 66.0±5.1	239/237	140/122	20 mL 0.5% ropivacaine	20 mL 0.9% saline	①②⑤⑥
Eppel et al., 2023 [28]	Germany	Hip arthroscopy	General	Preoperative	PENG vs Sham	30.9±6.4 / 30.1±6.8	34/34	22/23	20 mL 0.375% ropivacaine	20 mL 0.9% saline	①②③
Kukreja et al., 2023 [19]	USA	Total hip arthroplasty	Spinal	Preoperative	PENG vs None	58.5±14.75 / 62.1±11.25	56/56	26/22	25 mL 0.5% bupivacaine	None	①②③⑥
Lin et al., 2023 [29]	Singapore	Hip fracture surgery	Spinal	Preoperative	PENG vs Sham	80.2±6.8 / 76.3±7.5	28/29	8/9	20 mL 0.5% ropivacaine	None (a blunt needle was used that did not penetrate the skin)	①②③

Values are n or mean ± SD unless otherwise noted.

PENG, pericapsular nerve group; ① Pain score on a visual analog scale; ② Opioid consumption within 24 h after surgery; ③ Nausea and vomiting; ④ Patient dissatisfaction; ⑤ Time to first opioid consumption; ⑥ Functional recovery

### 3.2 Risk of bias

**Fig 2:**
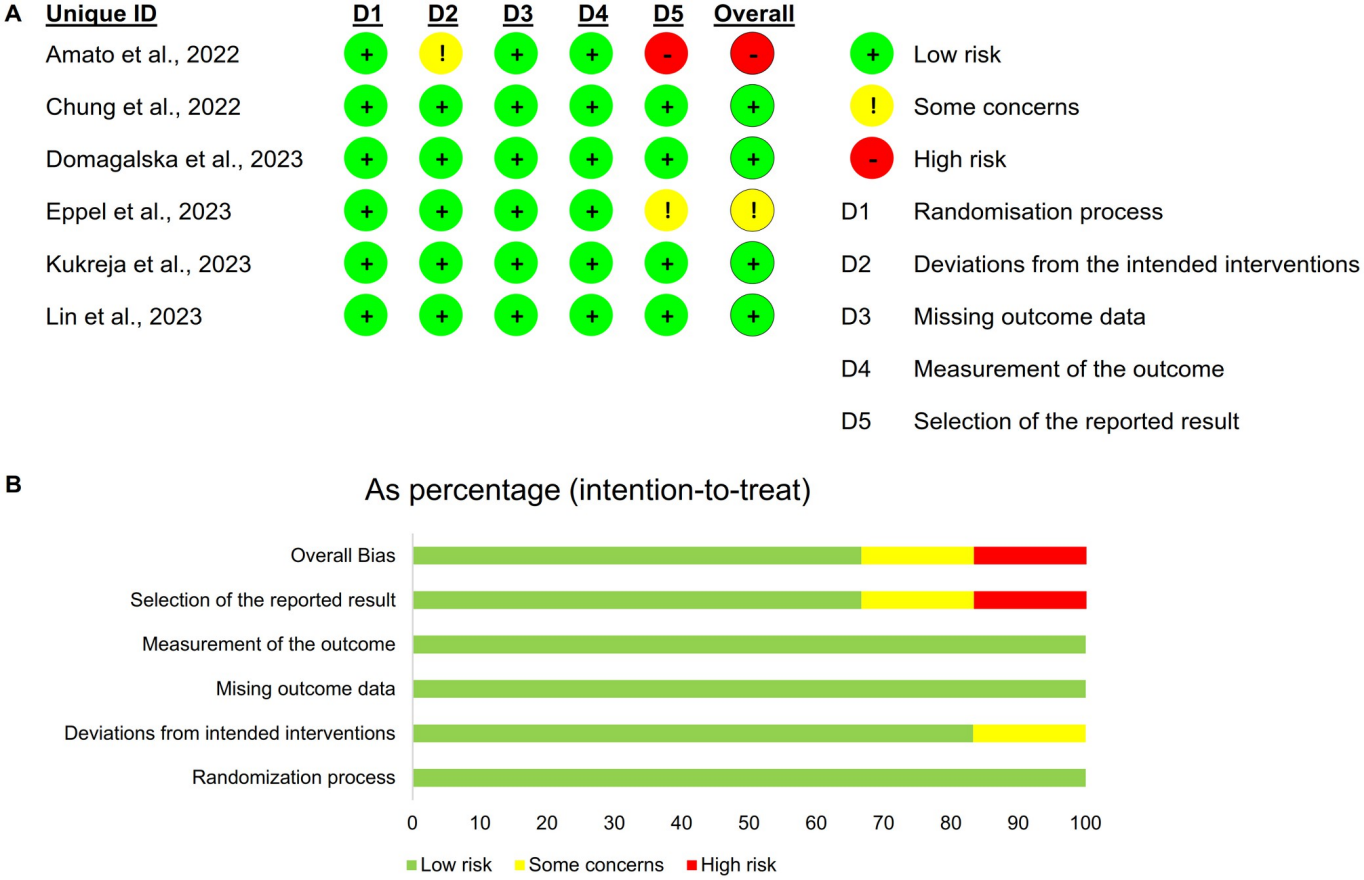
Risk of bias for the included literature. A. Summary of risk of bias; B. Percentage of risk of bias.

We assessed four of the six RCTs as having a low risk of bias, one as having an unclear risk of bias because we were unable to access the study protocol [28], and one as having a high risk of bias because we identified deviations from the original protocol and risk of selective reporting [20] (Fig 2).

### 3.3 The primary outcomes of meta-analysis

We focused on opioid consumption and pain within 24 h postoperatively because in most patients undergoing hip surgery, acute severe pain disappears within 4–6 h [30].

#### 3.3.1 Opioid consumption within 24 h after surgery

**Fig 3:**
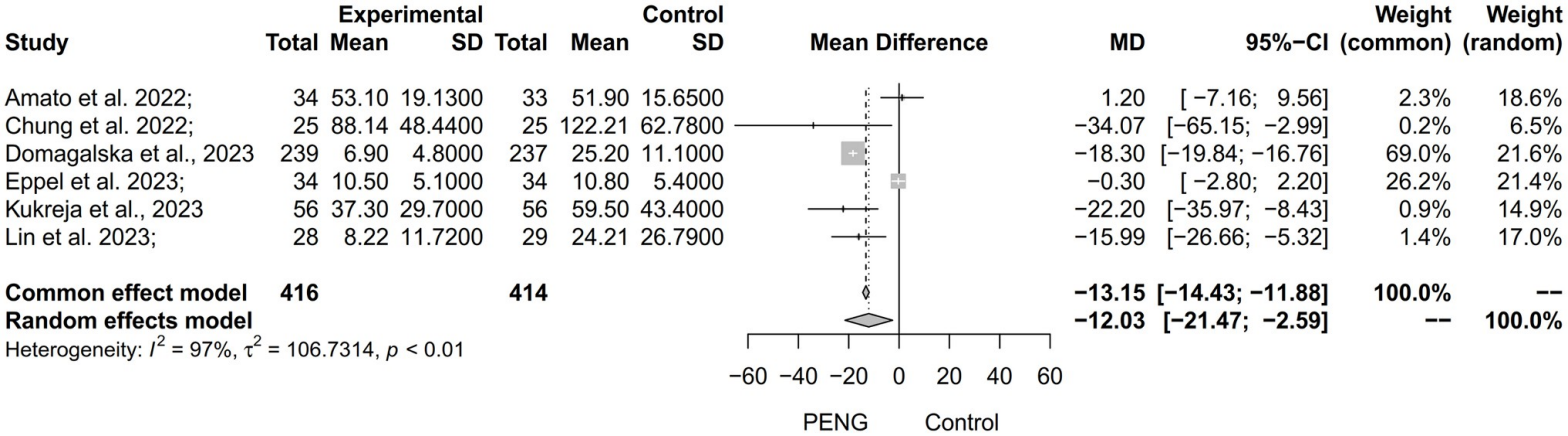
Forest plot of pooled results for opioid consumption within 24 h after hip surgery.

All six of the included RCTs examined opioid consumption within 24 h after surgery. PENG block was associated with significantly lower consumption (MD = -12.03, 95%CI: -21.47 to -2.59, *P* < 0.01, *I*^2^ = 97%; Fig 3). Furthermore, sensitivity analyses showed no significant differences in the pooled results after excluding Domagalska et al. [17] (MD = -10.56, 95%CI: -21.84 to 0.71, *P* = 0.07, *I*^*2*^ = 81%; Fig 1 of S5 Appendix).

**Fig 4:**
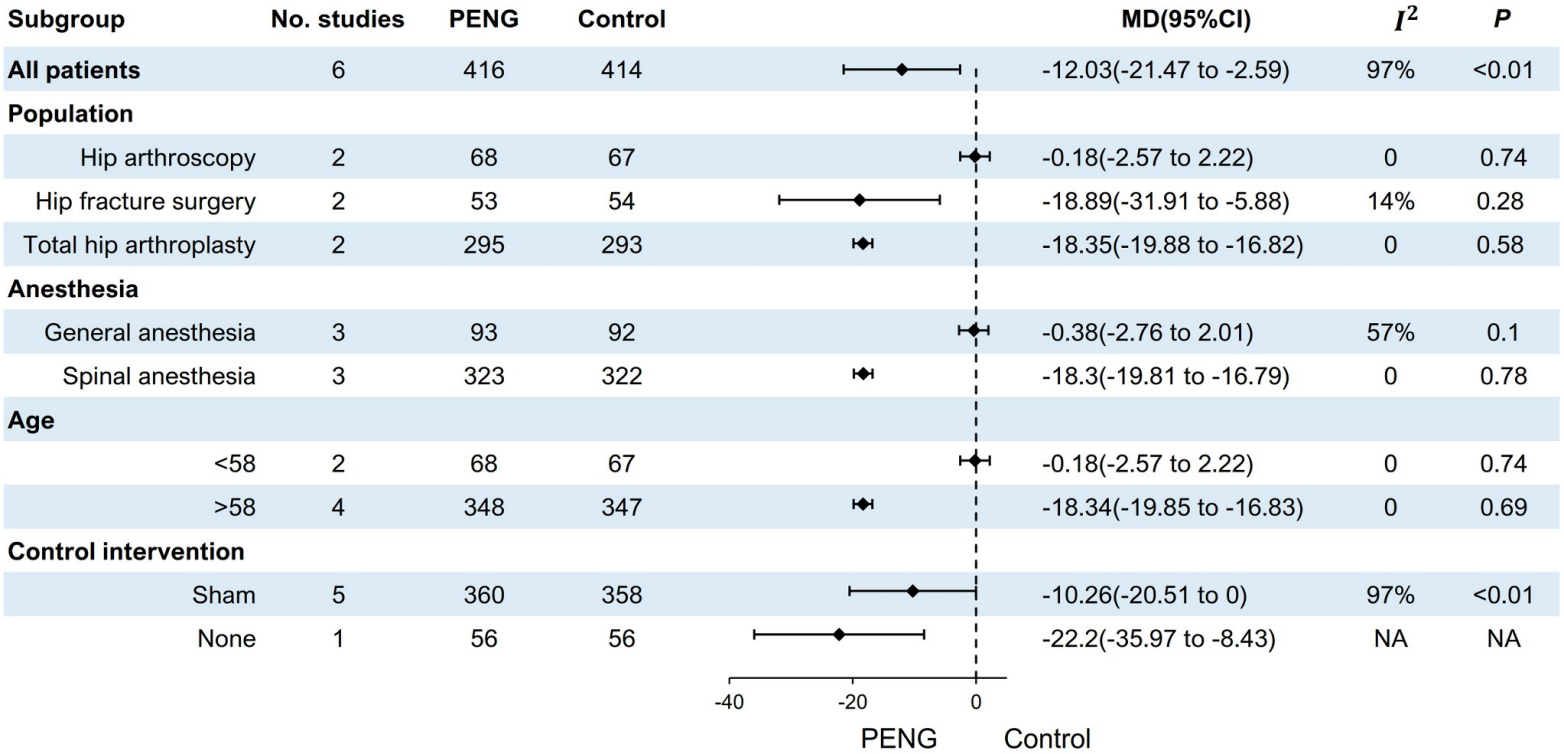
Forest plot of the subgroup analysis of opioid consumption within 24 h after hip surgery.

To investigate the source of heterogeneity, we conducted further subgroup analyses based on the population, anesthesia, age, and intervention in the control group (Fig 4). Subgroup analysis based on the population showed that PENG block was able to significantly reduce opioid consumption in hip fracture surgery (MD = -18.89, 95%CI: -31.91 to -5.88, *P* = 0.28, *I*^*2*^ = 14%) and total hip arthroplasty (MD = -18.35, 95%CI: -19.88 to -16.82, *P* = 0.58, *I*^*2*^ = 0%) populations with reduced heterogeneity in all groups (all *I*^*2*^ < 50%). Subgroup results based on anesthesia indicated that PENG block led to a significant reduction in opioid consumption among those who received spinal anesthesia (MD = -18.3, 95%CI: -19.81 to -16.79, *P* = 0.78, *I*^*2*^ = 0%), though there remained high heterogeneity within the general anesthesia group (*I*^*2*^ = 57%). Furthermore, a subgroup analysis based on age demonstrated increased benefits with PENG block for individuals aged above 58 years (MD = -18.34, 95%CI: -19.85 to -16.83, *P* = 0.69, *I*^*2*^ = 0%), and reduced heterogeneity was observed in both groups (all *I*^*2*^ = 0%). In contrast to the low heterogeneity observed in these analyses, subgroup analyses of control patients who received sham PENG block exhibited high levels of heterogeneity.

#### 3.3.2 Static pain scores

Five studies [17, 19, 20, 28, 29] reported static scores based on a visual analog scale within 24 h of surgery (Fig 2 of S5 Appendix). Within these five studies, three studies [19, 28, 29] involving 232 patients reported pain scores at 1 h after surgery, which did not differ significantly between the two groups (MD = -0.39, 95%CI: -0.86 to 0.08, *P* = 0.55, *I*^*2*^ = 0%). Two studies [28, 29] involving 111 patients reported scores at 3 h after surgery, but we could not meta-analyze them because one study [29] reported a score of 0 in the PENG group. Neither study reported a significant difference between the groups. Two studies [19, 28] involving 180 patients reported scores at 6 h after surgery, with no significant differences between the groups (MD = -0.27, 95%CI: -0.80 to 0.26, *P* = 0.87, *I*^*2*^ = 0%). Finally, four studies [17, 19, 20, 29] involving 685 patients found no significant difference between the two groups at 24 h after surgery (MD = -0.80, 95%CI: -2.29 to 0.70, *P* < 0.01, *I*^*2*^ = 95%), which proved robust to sensitivity analysis (Fig 3 of S5 Appendix).

#### 3.3.3 Dynamic pain scores

**Fig 5:**
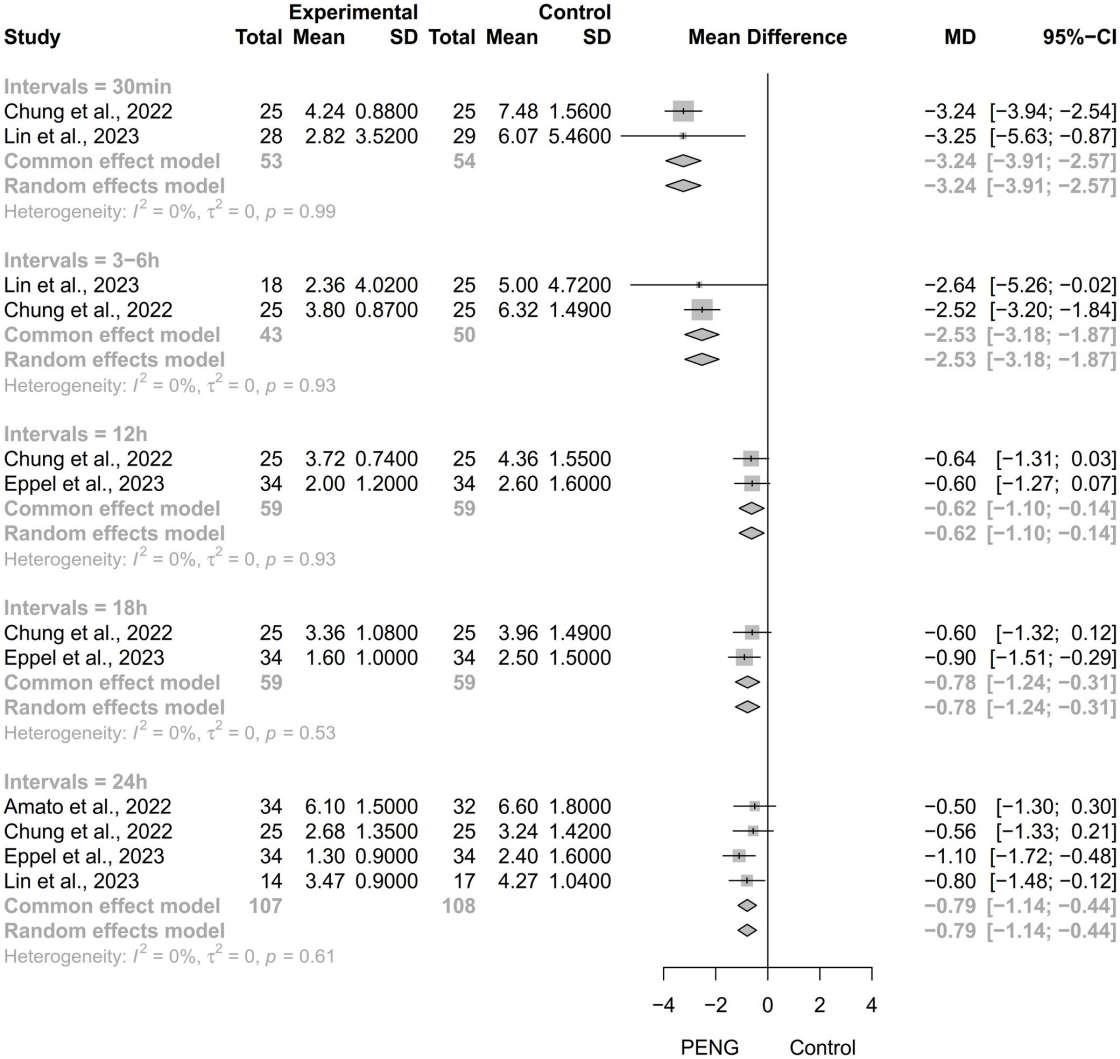
Forest plot of the dynamic pain score within 24 h after hip surgery, with monitoring at 30 min as well as 3–6, 12, 18, and 24 h postoperatively.

Four studies [20, 27–29] reported dynamic scores based on a visual analog scale at several time points within 24 h after surgery (Fig 5). Two trials [27, 29] involving 104 patients reported that PENG block was associated with significantly lower scores at 30 min (MD = -3.24, 95%CI: -3.91 to -2.57, *P* = 0.99, *I*^*2*^ = 0%). The same two studies reported, for a total of 93 patients, that PENG block was associated with significantly lower scores at 3–6 h (MD = -2.53, 95%CI: -3.18 to -1.87, *P* = 0.93, *I*^*2*^ = 0%). Similar results were reported for scores at 12 h (MD = -0.62, 95%CI: -1.10 to -0.14, *P* = 0.93, *I*^*2*^ = 0%) in two studies [27, 28] involving 118 patients, for scores at 18 h (MD = -0.78, 95%CI: -1.24 to -0.31, *P* = 0.53, *I*^*2*^ = 0%) in two studies [27, 28] involving 118 patients, and for scores at 24 h (MD = -0.79, 95%CI: -1.14 to -0.44, *P* = 0.61, *I*^*2*^ = 0%) in four studies [20, 27–29] involving 215 patients. Notably, all *I*^2^-related *P* values were greater than 0.05, indicating low heterogeneity among the pooled studies.

### 3.4 Secondary outcomes

#### 3.4.1 Time to the first opioid requirement

Two studies involving 526 patients reported time to first opioid requirement, but in different ways that could not be meta-analyzed. One study [27] reported that all patients in the control group required opioids within 12 h after surgery, compared to only 32% of patients who received PENG block. The other study [17] reported that PENG block was associated with a significantly longer time to first opioid requirement (9.5 ± 5.5 vs. 4.5 ± 1.6 h).

#### 3.4.2 Functional recovery

Three studies [17, 19, 27] involving 638 patients reported that PENG block was associated with better functional recovery based on different metrics that could not be meta-analyzed. One study [27] found no significant difference in quadricep strength between the two groups at 6–24 h after surgery. Another study [17] reported that significantly higher proportions of PENG patients were able to actively elevate the operated limb at 6 h after surgery (53 vs. 11%) and walk at 10 h postoperatively (100 vs. 46%). The third study [19] associated PENG block with a significantly higher quality of recovery based on the QoR-15 survey [31] at 24–48 h after surgery, although mean walking distance did not differ significantly between the groups on days 1 and 2 after surgery.

#### 3.4.3 Nausea and vomiting

Meta-analysis of three studies [20, 27, 28] involving 184 patients indicated no significant difference between the two groups in the incidence of nausea or vomiting at 24 h after surgery (RR = 0.53, 95%CI: 0.20 to 1.38, *P* = 0.73, *I*^*2*^ = 0%; Fig 4A of S5 Appendix). Similar results were obtained for incidence at 48 h from the meta-analysis of two studies [19, 20] involving 174 patients (RR = 0.97, 95%CI: 0.59 to 1.58, *P* = 0.49, *I*^*2*^ = 0%; Fig 4B of S5 Appendix).

#### 3.4.4 Patient dissatisfaction

Meta-analysis of data from two studies [20, 27] involving 116 people associated PENG block with significantly lower risk of unsatisfactory events (RR = 0.22, 95%CI: 0.08 to 0.61, *P* = 0.70, *I*^*2*^ = 0%; Fig 5 of S5 Appendix).

### 3.5 Quality of meta-analytical evidence

The quality of evidence for the various outcomes that we analyzed was classified according to the GRADE system as very low for static pain scores at 24 h; moderate for static pain scores at other time points; moderate for dynamic pain scores at all time points; low for opioid consumption within 24 h, reflecting substantial heterogeneity in the meta-analyses and risk of bias in one study [20]; high for nausea and vomiting; and moderate for patient dissatisfaction (Table 2).

**Table 2:** Quality of meta-analytical evidence based on the GRADE system.

Outcome meta-analyzed	No. studies	Risk of bias	Inconsistency	Indirectness	Imprecision	Other considerations	No. patients, PENG/Control	Pooled effect as	Quality	Importance
								MD or RR (95%CI)		
**Pain score at rest within…. after surgery**										
**1 h**	3	Serious	Not serious	Not serious	Not serious	None	114/118	MD: -0.39 (-0.86, 0.08)	+++	CRITICAL
									Moderate	
**3 h**	2	Serious	Not serious	Not serious	Not serious	None	52/59	Not done	+++	CRITICAL
									Moderate	
**6 h**	2	Serious	Not serious	Not serious	Not serious	None	90/90	MD: -0.27 (-0.80, 0.26)	+++	CRITICAL
									Moderate	
**24 h**	4	Very serious	Serious	Not serious	Serious	None	343/342	MD: -0.80 (-2.29, 0.70)	+	CRITICAL
									Very low	
**Pain score in motion within…. after surgery**										
**30 min**	2	Not serious	Not serious	Not serious	Serious	None	53/54	MD: -3.24 (-3.91, -2.57)	+++	CRITICAL
									Moderate	
**3–6 h**	2	Not serious	Not serious	Not serious	Serious	None	43/50	MD: -2.53 (-3.18, -1.87)	+++	CRITICAL
									Moderate	
**12 h**	2	Serious	Not serious	Not serious	Not serious	None	59/59	MD: -0.62 (-1.10, -0.14)	+++	CRITICAL
									Moderate	
**18 h**	2	Serious	Not serious	Not serious	Not serious	None	59/59	MD: -0.78 (-1.24, -0.31)	+++	CRITICAL
									Moderate	
**24 h**	4	Serious	Not serious	Not serious	Not serious	None	107/108	MD: -0.79 (-1.14, -0.44)	+++	CRITICAL
									Moderate	
**Opioid consumption within 24 h**	6	Serious	Serious	Not serious	Serious	None	416/414	MD: -12.03 (-21.47, -2.59)	+	CRITICAL
									Very low	
**Patient dissatisfaction**	2	Serious	Not serious	Not serious	Not serious	None	59/57	RR: 0.22 (0.08, 0.61)	+++	IMPORTANT
									Moderate	
**Nausea and vomiting**	3	Not serious	Not serious	Not serious	Not serious	None	115/115	RR: 0.88 (0.53, 1.46)	++++	IMPORTANT
									High	

CI, confidence interval; GRADE, Grading of Recommendations Assessment, Development, and Evaluation System [25]; MD, mean difference; PENG, pericapsular nerve group; RR, risk ratio.

## 4. Discussion

Although several meta-analyses [32–35] have examined the differences between PENG and other analgesic techniques in pain relief and postoperative recovery after hip surgery, there is still a lack of a relevant meta-analysis comparing PENG directly with sham/no block. Another meta-analysis of five randomized controlled trials [36] concluded that the PENG block provided better pain control than sham/no block. Still, this analysis may be less reliable as it pooled data from patients who received local anesthetic infiltration. This meta-analysis, including the most recent studies comparing PENG block against sham or no block, provides strong evidence that preoperative PENG block effectively reduces dynamic pain, although not necessarily static pain, soon after different types of hip surgery. The PENG block may also prolong the time before opioids are first needed after surgery and it may improve early functional recovery, but further studies are required to verify these possibilities. Most of our meta-analyses provide evidence of only moderate quality and some provide evidence of very low quality, highlighting the need for more rigorous assessments of the efficacy of the PENG block. Nevertheless, the evidence already available supports the PENG block as an effective tool in protocols designed to enhance recovery after surgery [37].

While the PENG block reduced opioid consumption across the various types of hip surgery in the included RCTs, it did not reduce consumption in the subgroup of patients who underwent hip arthroscopy. This procedure is less invasive and therefore intrinsically less likely to cause substantial pain, so any effect of the PENG block may be less noticeable. This may also explain why we failed to observe the efficacy of the PENG block in patients younger than 58 years old: both studies in that subgroup analysis involved hip arthroscopy [20, 28]. Subgroup analysis also showed that the PENG block reduced opioid consumption in patients receiving spinal, but not general, anesthesia. This may reflect that pain within 24 h after hip surgery is usually greater in patients who received spinal anesthesia than in those who received general anesthesia [38], which may translate to greater perceived efficacy of the PENG block. While opioid consumption within 24 hours of hip surgery was statistically significant, clinicians should carefully consider this when making clinical decisions given the included studies and small sample sizes.

Our failure to observe the benefit of the PENG block on static pain scores may reflect low static pain in the patient populations, such that the analgesic efficacy of the PENG block was less perceptible. A similar lack of benefit in static, but not dynamic, pain has been observed in individual studies [18, 39]. Even if our meta-analysis of included studies showed no benefit of PENG block on static pain, the data from individual studies consistently showed some positive effects. It is worth noting that while dynamic pain scores showed differences between the PENG and control groups at 12, 18, and 24 hours postoperatively, these differences were minimal, being less than one point. Such minor variations are unlikely to represent a clinically significant difference in the assessment of pain.

Two studies in our review reported that the PENG block prolonged the time to the first opioid requirement, but we could not meta-analyze these data because of differences in the way outcomes were reported. Other studies suggest that this effect of PENG block may be amplified by combining it with local infiltration anesthesia [18, 40], which should be explored in future work.

Two studies in our review reported that PENG block significantly improved functional recovery, while a third study failed to detect benefit. Such failure may reflect diffusion of local anesthetic into the femoral nerve [41]. Future work should examine this possibility and explore under what conditions the PENG block can enhance recovery. Also, nausea and vomiting events should be considered with caution due to the small number of included studies and sample sizes.

It should be noted that this study is subject to certain limitations. Firstly, the study included only six eligible RCTs, and the results were rated as moderate- or low-quality evidence using the GRADE Level of Evidence score. Therefore, the findings should be interpreted with caution. It would be beneficial to conduct higher-quality RCTs in the future to support the conclusions drawn from this study. Secondly, although subgroup analyses were conducted based on the type of surgery, anesthesia, age, and type of control intervention, the effects on pain may still be a source of high heterogeneity due to the inclusion of participants with a wide age range [42] and from different ethnic backgrounds [43]. It is recommended that future studies should endeavor to control the effect of confounders on outcomes as much as possible. Thirdly, before pooling the target endpoints, we attempted to standardize the same endpoint across studies to the greatest extent possible. However, given the subjective nature of pain, the different routes of opioid administration, and other factors, there may still be discrepancies in the same endpoint after standardization across studies. Fourthly, for outcomes such as time to first opioid requirement and functional recovery, different studies inconsistently presented them. Consequently, this study only provided a qualitative description. At last, our investigation did not delve into additional adverse effects of opioid utilization, encompassing sedation and delirium, nor did it scrutinize complications pertaining to the PENG block, such as vascular puncture and infection, owing to their infrequent documentation in the preliminary studies. Consequently, it is imperative for future endeavors, encompassing larger sample sizes, to strive towards a more comprehensive assessment of the safety profile associated with the PENG block.

## 5. Conclusion

The evidence available from RCTs suggests that PENG block significantly reduces opioid consumption and pain soon after hip surgery and may also improve early functional recovery. However, the limited number of included studies and sample size make it difficult to draw firm conclusions. Future studies are needed to support this study’s results and clarify the efficacy of PENG block according to patient age, surgery, and anesthesia, which clinicians may need to consider when choosing multimodal analgesia.

## Supporting information

S1 Appendix PRISMA checklist. (DOCX)

S2 Appendix Search strategy. (DOCX)

S3 Appendix Data extracted from studies. (DOCX)

S4 Appendix List of excluded studies with reasons and included studies. (DOCX)

S5 Appendix Forest plots those were not included in the manuscript. (DOCX)

S1 Raw data (DOCX)

## Abbreviations

95%CI: 95% confidence intervals
GRADE: Grading of Recommendations Assessment, Development and Evaluation
MD: Mean difference
PENG: Pericapsular nerve group
PRISMA: Preferred Reporting Items for Systematic Reviews and Meta-Analyses
QoR-15: Quality of Recovery-15 score
RCTs: Randomized controlled trials
RR: Risk ratio
