# Supplementary material for: Pericapsular nerve group block reduces opioid use and pain after hip surgery: A systematic review and meta-analysis of randomized controlled trials
Source: PLoS One. 2024 Nov 8;19(11):e0310008. doi: 10.1371/journal.pone.0310008 (PMC11548832; doi:10.1371/journal.pone.0310008)
Supplement: S2 Appendix — (DOCX) [file pone.0310008.s002.docx]

**Search strategy**

**Pubmed**

((("Hip"[Mesh]) OR (((((((((((((((((((((hips) OR (Hip surgery)) OR (Hip fracture surgery)) OR (Hip fracture fixation)) OR (Total hip arthroplasty)) OR (THA)) OR (Total hip replacement)) OR (THR)) OR (Hemiarthroplasty)) OR (Hip arthroscopy)) OR (Internal Fixation)) OR (Screw fixation)) OR (Compression hip screw)) OR (Proximal Femoral Nail Antirotation)) OR (PFNA)) OR (Proximal Femoral Nail)) OR (PFN)) OR (Dynamic Hip Screw)) OR (DHS)) OR (Gamma nail)) OR (External fixation))) AND (((Pericapsular nerve group) OR (PENG)) OR (Pericapsul*))) AND (("Placebos"[Mesh]) OR (((((Placebo) OR (Sham)) OR ("No block")) OR (Saline)) OR (NaCl)))

**Results:46**

**Embase**

| No. | Query | Results |
| --- | --- | --- |
| #4 | #1 AND #2 AND #3 | 36 |
| #3 | 'placebos':ab,ti OR 'placebo':ab,ti OR 'sham':ab,ti OR 'no block':ab,ti OR 'saline':ab,ti OR 'nacl':ab,ti | 853523 |
| #2 | 'pericapsular nerve group':ab,ti OR 'peng':ab,ti OR 'pericapsul*':ab,ti | 2726 |
| #1 | 'hip':ab,ti OR 'hips':ab,ti OR 'hip surgery':ab,ti OR 'hip fracture surgery':ab,ti OR 'hip fracture fixation':ab,ti OR 'total hip arthroplasty':ab,ti OR 'tha':ab,ti OR 'total hip replacement':ab,ti OR 'thr':ab,ti OR 'hemiarthroplasty':ab,ti OR 'hip arthroscopy':ab,ti OR 'internal fixation':ab,ti OR 'screw fixation':ab,ti OR 'compression hip screw':ab,ti OR 'proximal femoral nail antirotation':ab,ti OR 'pfna':ab,ti OR 'proximal femoral nail':ab,ti OR 'pfn':ab,ti OR 'dynamic hip screw':ab,ti OR 'dhs':ab,ti OR 'gamma nail':ab,ti OR 'external fixation':ab,ti | 324506 |

**Results:36**

**Cochrane Library**

| ID | Search | Results |
| --- | --- | --- |
| #1 | (Hip):ti,ab,kw OR (hips):ti,ab,kw OR (Hip surgery):ti,ab,kw OR (Hip fracture surgery):ti,ab,kw OR (Hip fracture fixation):ti,ab,kw OR (Total hip arthroplasty):ti,ab,kw OR (THA):ti,ab,kw OR (Total hip replacement):ti,ab,kw OR (THR):ti,ab,kw OR (Hemiarthroplasty):ti,ab,kw OR (Hip arthroscopy):ti,ab,kw OR (Internal Fixation):ti,ab,kw OR (Screw fixation):ti,ab,kw OR (Compression hip screw):ti,ab,kw OR (Proximal Femoral Nail Antirotation):ti,ab,kw OR (PFNA):ti,ab,kw OR (Proximal Femoral Nail):ti,ab,kw OR (PFN):ti,ab,kw OR (Dynamic Hip Screw):ti,ab,kw OR (DHS):ti,ab,kw OR (Gamma nail):ti,ab,kw OR (External fixation):ti,ab,kw | 37078 |
| #2 | (Pericapsular nerve group):ti,ab,kw OR (PENG):ti,ab,kw OR (Pericapsul*):ti,ab,kw | 553 |
| #3 | (Placebos):ti,ab,kw OR (Placebo):ti,ab,kw OR (Sham):ti,ab,kw OR (No block):ti,ab,kw OR (Saline):ti,ab,kw OR (NaCl):ti,ab,kw | 458811 |
| #4 | #1 and #2 and #3 | 154 |

**Results:154**

**Web of Science**

| Set | Results | Search history |
| --- | --- | --- |
| #4 | 102 | #1 AND #2 AND #3 |
| #3 | 1335590 | TS=(Placebos or Placebo or Sham or No block or Saline or NaCl) |
| #2 | 10735 | TS=(Pericapsular nerve group or PENG or Pericapsul*) |
| #1 | 517198 | TS=(Hip or hips or Hip surgery or Hip fracture surgery or Hip fracture fixation or Total hip arthroplasty or THA or Total hip replacement or THR or Hemiarthroplasty or Hip arthroscopy or Internal Fixation or Screw fixation or Compression hip screw or Proximal Femoral Nail Antirotation or PFNA or Proximal Femoral Nail or PFN or Dynamic Hip Screw or DHS or Gamma nail or External fixation) |

**Results:102**
