# Supplementary material for: Pericapsular nerve group block reduces opioid use and pain after hip surgery: A systematic review and meta-analysis of randomized controlled trials
Source: PLoS One. 2024 Nov 8;19(11):e0310008. doi: 10.1371/journal.pone.0310008 (PMC11548832; doi:10.1371/journal.pone.0310008)
Supplement: S3 Appendix — (DOCX) [file pone.0310008.s003.docx]

**Data extracted from studies.**

**1. Opioid consumption within 24 h after surgery**

**2. Static pain scores within 24 h of surgery**

**3. Dynamic pain scores within 24 h of surgery**

**4. Nausea and vomiting**

**5. Patient dissatisfaction**

| **1. Opioid consumption within 24 h after surgery** | | | | | | | | | | |
| --- | --- | --- | --- | --- | --- | --- | --- | --- | --- | --- |
| study | n1 | mean1 | sd1 | n2 | mean2 | sd2 | Population | Anesthesia | Age | C.Intervention |
| Amato et al., 2022 | 34 | 53.1 | 19.13 | 33 | 51.9 | 15.65 | arthroscopic hip surgery | General anesthesia | <40 | Placebo |
| Chung et al., 2022 | 25 | 88.14 | 48.44 | 25 | 122.21 | 62.78 | hip surgery | General anesthesia | >40 | Placebo |
| Domagalska et al., 2023 | 239 | 6.9 | 4.8 | 237 | 25.2 | 11.1 | THA | Spinal anesthesia | >40 | Placebo |
| Eppel et al., 2023 | 34 | 10.5 | 5.1 | 34 | 10.8 | 5.4 | arthroscopic hip surgery | General anesthesia | <40 | Placebo |
| Kukreja et al., 2023 | 56 | 37.3 | 29.7 | 56 | 59.5 | 43.4 | THA | Spinal anesthesia | >40 | None |
| Lin et al., 2023 | 28 | 8.22 | 11.72 | 29 | 24.21 | 26.79 | hip surgery | Spinal anesthesia | >40 | Placebo |

| **2. Static pain scores within 24 h of surgery** | | | | | | | | |
| --- | --- | --- | --- | --- | --- | --- | --- | --- |
| study | Intervals | n1 | mean1 | sd1 | n2 | mean2 | sd2 | Intervals |
| Eppel et al., 2023 | 1h/PACU | 34 | 3.4 | 1.6 | 34 | 3.6 | 1.7 | 1h |
| Kukreja et al., 2023 | 1h/PACU | 56 | 1.8 | 1.5 | 56 | 2.3 | 1.7 | PACU |
| Lin et al., 2023 | 1h/PACU | 24 | 0 | 0 | 28 | 0 | 0 | 1h |
| Eppel et al., 2023 | 3h | 34 | 2.9 | 1.4 | 34 | 3.1 | 1.5 | 3h |
| Lin et al., 2023 | 3h | 18 | 0 | 0 | 25 | 0.16 | 0.38 | 3h |
| Eppel et al., 2023 | 6h | 34 | 2.4 | 1.3 | 34 | 2.7 | 1.3 | 6h |
| Kukreja et al., 2023 | 6h | 56 | 2.3 | 2.7 | 56 | 2.5 | 2.8 | 6h |
| Amato et al., 2022 | 24h | 34 | 4 | 1.8 | 32 | 4 | 2.4 | 24h |
| Domagalska et al., 2023 | 24h | 239 | 3.1 | 1 | 237 | 5.3 | 1 | 24h |
| Kukreja et al., 2023 | 24h | 56 | 2.8 | 2.3 | 56 | 2.8 | 2.2 | 24h |
| Lin et al., 2023 | 24h | 14 | 0 | 0 | 17 | 0.05 | 0.1 | 24h |

| **3. Dynamic pain scores within 24 h of surgery** | | | | | | | |
| --- | --- | --- | --- | --- | --- | --- | --- |
| study | Intervals | n1 | mean1 | sd1 | n2 | mean2 | sd2 |
| Chung et al., 2022 | 30min | 25 | 4.24 | 0.88 | 25 | 7.48 | 1.56 |
| Lin et al., 2023 | 30min | 28 | 2.82 | 3.52 | 29 | 6.07 | 5.46 |
| Lin et al., 2023 | 3-6h | 18 | 2.36 | 4.02 | 25 | 5 | 4.72 |
| Chung et al., 2022 | 3-6h | 25 | 3.8 | 0.87 | 25 | 6.32 | 1.49 |
| Chung et al., 2022 | 12h | 25 | 3.72 | 0.74 | 25 | 4.36 | 1.55 |
| Eppel et al., 2023 | 12h | 34 | 2 | 1.2 | 34 | 2.6 | 1.6 |
| Chung et al., 2022 | 18h | 25 | 3.36 | 1.08 | 25 | 3.96 | 1.49 |
| Eppel et al., 2023 | 18h | 34 | 1.6 | 1 | 34 | 2.5 | 1.5 |
| Amato et al., 2022 | 24h | 34 | 6.1 | 1.5 | 32 | 6.6 | 1.8 |
| Chung et al., 2022 | 24h | 25 | 2.68 | 1.35 | 25 | 3.24 | 1.42 |
| Eppel et al., 2023 | 24h | 34 | 1.3 | 0.9 | 34 | 2.4 | 1.6 |
| Lin et al., 2023 | 24h | 14 | 3.47 | 0.9 | 17 | 4.27 | 1.04 |

| **4. Nausea and vomiting** | | | | |
| --- | --- | --- | --- | --- |
| 24 h | | | | |
| study | n1 | event1 | n2 | event2 |
| Amato et al., 2022 | 34 | 3 | 32 | 7 |
| Chung et al., 2022 | 25 | 2 | 25 | 2 |
| Eppel et al., 2023 | 34 | 1 | 34 | 2 |
| 48 h | | | | |
| study | n1 | event1 | n2 | event2 |
| Amato et al., 2022 | 32 | 6 | 30 | 4 |
| Kukreja et al., 2023 | 56 | 17 | 56 | 19 |

| **5. Patient dissatisfaction** | | | | |
| --- | --- | --- | --- | --- |
| study | n1 | event1 | n2 | event2 |
| Amato et al. 2022; | 34 | 1 | 32 | 6 |
| Chung et al. 2022; | 25 | 3 | 25 | 12 |
