# Supplementary material for: Pericapsular nerve group block reduces opioid use and pain after hip surgery: A systematic review and meta-analysis of randomized controlled trials
Source: PLoS One. 2024 Nov 8;19(11):e0310008. doi: 10.1371/journal.pone.0310008 (PMC11548832; doi:10.1371/journal.pone.0310008)
Supplement: S4 Appendix — (DOCX) [file pone.0310008.s004.docx]

**List of excluded studies with reasons (n=261) and included studies (n=6).**

1. **Records excluded by type of work (n=162).**
   1. **Experimental registries (n=95)**
   2. **Conference abstracts (n=34)**
   3. **Reviews (n=23)**
   4. **Others (n=10)**
2. **Records excluded by titles and abstracts (n=90)**
   1. **Unrelated topics (n=33)**
   2. **Comparison with other analgesic techniques (n=31)**
   3. **Non-randomized control studies (n=18)**
   4. **Case Report (n=8)**
3. **Full-text articles excluded, with reasons (n=9)**
   1. **Combined with other analgesic techniques (n=7)**
   2. **Other reasons(n=2)**
4. **Studies eventually included (n=6)**

**1. Records excluded by type of work (n=162).**

**1.1 Experimental registries (n=95)**

| **No.** | **Author and year** | **Reasons of exclusion** |
| --- | --- | --- |
| 1 | Abdelrhman Alshawadfy, Suez Canal University. 2021 | Experimental registries |
| 2 | Ahmad 2023 | Experimental registries |
| 3 | Albers 2021 | Experimental registries |
| 4 | Albrecht 2024 | Experimental registries |
| 5 | Altinsoy 2023 | Experimental registries |
| 6 | Amato 2020 | Experimental registries |
| 7 | Likourezos 2021 | Experimental registries |
| 8 | Likourezos 2021 | Experimental registries |
| 9 | Ayyan 2023 | Experimental registries |
| 10 | Ciftci 2021 | Experimental registries |
| 11 | Bhatia 2023 | Experimental registries |
| 12 | Bozyaka Training and Research Hospital. 2023 | Experimental registries |
| 13 | Bugada 2023 | Experimental registries |
| 14 | Bugada 2023 | Experimental registries |
| 15 | Cardoso 2023 | Experimental registries |
| 16 | Carella 2021 | Experimental registries |
| 17 | Carella 2022 | Experimental registries |
| 18 | Centre hospitalier de l'Université de Montréal. 2020 | Experimental registries |
| 19 | Centre hospitalier de l'Université de Montréal. 2020 | Experimental registries |
| 20 | Chan 2021 | Experimental registries |
| 21 | Chand 2024 | Experimental registries |
| 22 | Chen et al., 2023 | Experimental registries |
| 23 | Cheok 2023 | Experimental registries |
| 24 | Chethan et al., 2024 | Experimental registries |
| 25 | Ciftci 2022 | Experimental registries |
| 26 | Dhir 2023 | Experimental registries |
| 27 | Duan 2020 | Experimental registries |
| 28 | Eldine et al., 2022 | Experimental registries |
| 29 | Eloy 2012 | Experimental registries |
| 30 | Gomez 2023 | Experimental registries |
| 31 | Hanoi Medical University. 2023 | Experimental registries |
| 32 | Haseki Training and Research Hospital. 2024 | Experimental registries |
| 33 | Haseki Training and Research Hospital. 2024 | Experimental registries |
| 34 | Helmy 2023 | Experimental registries |
| 35 | Hisham mohamed gamal eldine hassan, Alexandria University. 2022 | Experimental registries |
| 36 | Hong et al., 2022 | Experimental registries |
| 37 | Huang et al., 2022 | Experimental registries |
| 38 | Ilfeld 2021 | Experimental registries |
| 39 | Ismail 2022 | Experimental registries |
| 40 | Ital 2023 | Experimental registries |
| 41 | Jin et al., 2022 | Experimental registries |
| 42 | Jyothi 2023 | Experimental registries |
| 43 | Kaabachi 2020 | Experimental registries |
| 44 | Kalashetty et al., 2023 | Experimental registries |
| 45 | Kang 2022 | Experimental registries |
| 46 | Khandelwal et al., 2023 | Experimental registries |
| 47 | Kukreja 2020 | Experimental registries |
| 48 | Kumar 2020 | Experimental registries |
| 49 | Kumar et al., 2024 | Experimental registries |
| 50 | Lai 2022 | Experimental registries |
| 51 | Lee et al., 2021 | Experimental registries |
| 52 | Li et al., 2021 | Experimental registries |
| 53 | Li et al., 2023 | Experimental registries |
| 54 | LifeBridge Health. 2020 | Experimental registries |
| 55 | Lin 2020 | Experimental registries |
| 56 | Lin 2020 | Experimental registries |
| 57 | Lin 2020 | Experimental registries |
| 58 | Lin 2020 | Experimental registries |
| 59 | Lin 2020 | Experimental registries |
| 60 | Lin 2021 | Experimental registries |
| 61 | Meierhernich 2024 | Experimental registries |
| 62 | Mitra 2021 | Experimental registries |
| 63 | Morales-Avalos 2021 | Experimental registries |
| 64 | Mostafa 2020 | Experimental registries |
| 65 | Mulier 2018 | Experimental registries |
| 66 | National University of Malaysia. 2023 | Experimental registries |
| 67 | Nuthep 2022 | Experimental registries |
| 68 | Panjiar 2023 | Experimental registries |
| 69 | Park et al., 2021 | Experimental registries |
| 70 | Pattnaik et al., 2024 | Experimental registries |
| 71 | Poznan University of Medical Sciences. 2023 | Experimental registries |
| 72 | Region Skane. 2024 | Experimental registries |
| 73 | Ruan 2020 | Experimental registries |
| 74 | Sahoo 2022 | Experimental registries |
| 75 | Samadpour 2020 | Experimental registries |
| 76 | Sameh Abdelkhalik Ahmed Ismaiel, Tanta University. 2021 | Experimental registries |
| 77 | Satici 2023 | Experimental registries |
| 78 | Seraglio 2021 | Experimental registries |
| 79 | Sidhu 2020 | Experimental registries |
| 80 | Singapore General Hospital. 2021 | Experimental registries |
| 81 | Sonawane 2019 | Experimental registries |
| 82 | Sujarittham 2019 | Experimental registries |
| 83 | Tah et al., 2021 | Experimental registries |
| 84 | Tsai 2021 | Experimental registries |
| 85 | Uludag 2023 | Experimental registries |
| 86 | University Hospital, Toulouse. 2020 | Experimental registries |
| 87 | University Hospital, Toulouse. 2020 | Experimental registries |
| 88 | Vardhan 2024 | Experimental registries |
| 89 | Vested M.2024 | Experimental registries |
| 90 | Wake Forest University Health Sciences. 2022 | Experimental registries |
| 91 | Wang 2023 | Experimental registries |
| 92 | Wang et al., 2021 | Experimental registries |
| 93 | Yonsei University. 2022 | Experimental registries |
| 94 | Yörükoğlu 2024 | Experimental registries |
| 95 | Zheng et al., 2024 | Experimental registries |

**References**

1. Abdelrhman Alshawadfy, Suez Canal University. Comparison Between Pericapsular Nerve Group Block (PENG) and Morphine Infusion https://clinicaltrialsgov/show/NCT05023473. 2021. PubMed PMID: CN-02307406.
2. Ahmad A. Efficacy of Pericapsular Nerve Group (PENG) Block for Hip Surgeries https://clinicaltrialsgov/ct2/show/NCT06144931. 2023. PubMed PMID: CN-02628557.
3. Albers A. Pain Control Following Total Hip Arthroplasty https://clinicaltrialsgov/ct2/show/NCT05062356. 2021. PubMed PMID: CN-02340929.
4. Albrecht E. Pericapsular Nerve Block Versus Intrathecal Morphine for Analgesia After Primary Hip Arthroplasty https://clinicaltrialsgov/ct2/show/NCT06317870. 2024. PubMed PMID: CN-02673075.
5. Altinsoy S. Evaluation of PENG Block in Terms of Block Time and Postoperative Pain https://clinicaltrialsgov/ct2/show/NCT06132308. 2023. PubMed PMID: CN-02626755.
6. Amato P. PENG Block for Arthroscopic Hip Surgery https://clinicaltrialsgov/show/NCT04508504. 2020. PubMed PMID: CN-02137928.
7. Likourezos A. PENG vs FIC Block for Hip Fracture ED Patients https://clinicaltrialsgov/ct2/show/NCT05102760. 2021. PubMed PMID: CN-02341433.
8. Likourezos A. PENG vs FIC Block for Hip Fracture ED Patients https://clinicaltrialsgov/show/NCT05102760. 2021. PubMed PMID: CN-02341433.
9. Ayyan M. Alternate procedure to the conventional treatment which will decrease the pain and discomfort in the patients presenting to department of emergency medicine with Hip injuries https://trialsearchwhoint/Trial2aspx?TrialID=CTRI/2023/08/056595. 2023. PubMed PMID: CN-02591691.
10. Ciftci B. Fascia Iliaca Compartment Block and PENG Block for Hip Arthroplasty https://clinicaltrialsgov/show/NCT05030688. 2021. PubMed PMID: CN-02307600.
11. Bhatia R. Ultrasound-guided pericapsular nerve group block following hip arthroplasty for postoperative analgesia https://trialsearchwhoint/Trial2aspx?TrialID=CTRI/2023/10/058678. 2023. PubMed PMID: CN-02625585.
12. Bozyaka Training and Research Hospital. Comparison of the Effects of Pericapsular Nerve Group (PENG), Suprainguinal Fascia Iliaca Compartment (S-FICB) and 3-1 Blocks on Comfort in Positioning for Unilateral Spinal Anesthesia and Postoperative Pain in Hip Fracture Surgery https://clinicaltrialsgov/show/NCT05912101. 2023. PubMed PMID: CN-02577553.
13. Bugada D. Erector Spinae Block Versus PENG Block for Hip Replacement https://clinicaltrialsgov/ct2/show/NCT05796804. 2023. PubMed PMID: CN-02540123.
14. Bugada D. Erector Spinae Block Versus PENG Block for Hip Replacement https://clinicaltrialsgov/show/NCT05796804. 2023. PubMed PMID: CN-02540123.
15. Cardoso FA. Evaluation of pain and inflammation of the PENG block associated with the femoral lateral cutaneous nerve block in hip prosthesis surgeries https://trialsearchwhoint/Trial2aspx?TrialID=RBR-86m8dm9. 2023. PubMed PMID: CN-02592083.
16. Carella M. Comparison between the PENG block and the supra-inguinal fascia iliaca compartment block on postoperative pain, total opioids consumption and early motor recovery after total hip arthroplasty https://trialsearchwhoint/Trial2aspx?TrialID=EUCTR2020-005126-28-BE. 2021. PubMed PMID: CN-02377975.
17. Carella M. Equivalence study for a comparison of the impact on postoperative functional recovery in total hip arthroplasty between periarticular infiltration, pericapsular nerve group block (PENG), and suprainguinal iliaca fascia block https://trialsearchwhoint/Trial2aspx?TrialID=EUCTR2022-002250-97-BE. 2022. PubMed PMID: CN-02513082.
18. Centre hospitalier de l'Université de Montréal. Effectiveness of PENG Block Combined to LFCN Block on the Quality of Recovery After Total Hip Replacement https://clinicaltrialsgov/ct2/show/NCT04245280. 2020. PubMed PMID: CN-02079815.
19. Centre hospitalier de l'Université de Montréal. Effectiveness of PENG Block Combined to LFCN Block on the Quality of Recovery After Total Hip Replacement https://clinicaltrialsgov/show/NCT04245280. 2020. PubMed PMID: CN-02079815.
20. Chan CW. Efficacy of Pericapsular Nerve Group Block https://clinicaltrialsgov/show/NCT05154318. 2021. PubMed PMID: CN-02353729.
21. Chand KN. Ultrasound guided(USG) pericapsular nerve group block (PENG) using 025% Bupivaicaine with or without magnesium sulphate for positional comfort during regional block in hip fracture patients https://trialsearchwhoint/Trial2aspx?TrialID=CTRI/2024/06/068519. 2024. PubMed PMID: CN-02721805.
22. Chen ZH, Wang QS. Impact of high-volume pericapsular nerve group (PENG) block on postoperative analgesia and motor function recovery following total hip arthroplasty: a prospective, observer-blinded randomized controlled trial https://trialsearchwhoint/Trial2aspx?TrialID=ChiCTR2300077281. 2023. PubMed PMID: CN-02620968.
23. Cheok T. The Utility of Pericapsular Nerve Group (PENG) Block versus Intrathecal Morphine for Postoperative Analgesia in Anterior Approach Total Hip Arthroplasty: a Multicentre Triple Blinded Randomised Controlled Trial https://trialsearchwhoint/Trial2aspx?TrialID=ACTRN12623001309673. 2023. PubMed PMID: CN-02634659.
24. Chethan OS, Khandelwal M. Comparitive study of ropivacaine vs ropivacaine with dexamethasone in ultrasound guided nerve block in patients undergoing hip surgery for preoperative and postoperative pain https://trialsearchwhoint/Trial2aspx?TrialID=CTRI/2024/07/070130. 2024. PubMed PMID: CN-02732201.
25. Ciftci B. Iliopsoas Plane Block vs PENG Block for Hip Arthroplasty https://clinicaltrialsgov/ct2/show/NCT05397145. 2022. PubMed PMID: CN-02405497.
26. Dhir S. PENG Block vs Intraoperative Local Anesthetic Infiltration for Total Hip Arthroplasty https://clinicaltrialsgov/ct2/show/NCT05898581. 2023. PubMed PMID: CN-02573102.
27. Duan L. Clinical application of ultrasound-guided pericapsular nerve group (PENG) block in hip fracture surgery: a prospective, double-blind, randomized controlled trial https://trialsearchwhoint/Trial2aspx?TrialID=ChiCTR2000034821. 2020. PubMed PMID: CN-02184598.
28. Eldine HG, Elnaggar A. Comparing two different paediatric regional anesthesia techniques for postoperative analgesia after surgical correction of developmental dysplasia of the hip joint https://trialsearchwhoint/Trial2aspx?TrialID=PACTR202204798992849. 2022. PubMed PMID: CN-02412762.
29. Eloy JD. Effect of Gabapentin on Orthopedic Pain https://clinicaltrialsgov/show/NCT01546857. 2012. PubMed PMID: CN-02033201.
30. Gomez RS. Lateral Cutaneous Nerve and PENG Blocks Versus Suprainguinal Fascia Iliaca Block in Post-op Analgesia of Hip Fractures https://clinicaltrialsgov/show/NCT05749367. 2023. PubMed PMID: CN-02528694.
31. Hanoi Medical University. PENG Block and Lateral Femoral Cutaneous Nerve Block For Hip Replacement Surgery https://clinicaltrialsgov/ct2/show/NCT06040879. 2023. PubMed PMID: CN-02599658.
32. Haseki Training and Research Hospital. Pericapsular Nerve Block and Iliopsoas Fascial Plane Block in Hip Surgery https://clinicaltrialsgov/ct2/show/NCT06267716. 2024. PubMed PMID: CN-02680468.
33. Haseki Training and Research Hospital. the Pericapsular Nerve Group (PENG) and Suprainguinal Fascia Iliaca Blocks (SIFIB) in Elderly Patients https://clinicaltrialsgov/ct2/show/NCT06277648. 2024. PubMed PMID: CN-02680741.
34. Helmy A. Pericapsular Block Versus Trans Muscular Quadratus Lumborum Block in Patients Undergoing Correction of Hip Dysplasia https://clinicaltrialsgov/ct2/show/NCT06130761. 2023. PubMed PMID: CN-02626711.
35. Hisham mohamed gamal eldine hassan, Alexandria University. Efficacy and Safety of Quadratus Lumborum Block Versus Pericapsular Nerve Group Block in Pediatric Hip Surgery https://clinicaltrialsgov/show/NCT05349656. 2022. PubMed PMID: CN-02394079.
36. Hong KY, Gwak MS. Evaluation of analgesic effects of ultrasound-guided pericapsular nerve group (PENG) block combined with lateral femoral cutaneous nerve (LFCN) block in total hip replacement arthroplasty under spinal anesthesia https://trialsearchwhoint/Trial2aspx?TrialID=KCT0007959. 2022. PubMed PMID: CN-02519661.
37. Huang Y, Dong CS. Comparison of modified iliac fascia block and peripheral nerve block combined with local infiltration in total hip replacement https://trialsearchwhoint/Trial2aspx?TrialID=ChiCTR2200065321. 2022. PubMed PMID: CN-02569745.
38. Ilfeld BM. Pericapsular Nerve Group Block (PENG) for Hip Surgery https://clinicaltrialsgov/show/NCT05118620. 2021. PubMed PMID: CN-02352808.
39. Ismail AE. Postoperative Analgesic Effect of Two Peripheral Nerve Blocks for Hip Surgery in Pediatrics https://clinicaltrialsgov/show/NCT05348421. 2022. PubMed PMID: CN-02394060.
40. Ital I. Evaluation of the Effectiviness of Two Different Bupivacaine Concentrations of the Pericapsular Nerve Group (PENG) Block https://clinicaltrialsgov/show/NCT05921110. 2023. PubMed PMID: CN-02577768.
41. Jin Z, Sugiyama D. PENG block and pre-habilitation https://trialsearchwhoint/Trial2aspx?TrialID=JPRN-jRCT1031220294. 2022. PubMed PMID: CN-02513136.
42. Jyothi B. COMPARISON OF POSITIONING AFTER PENG BLOCK PRIOR TO SPINAL ANAESTHESIA FOR HIP SURGERIES FOLLOWING DIFFERENT VOLUMES OF ROPIVACAINE https://trialsearchwhoint/Trial2aspx?TrialID=CTRI/2023/06/053462. 2023. PubMed PMID: CN-02576543.
43. Kaabachi O. Pericapsular Nerve Group Block for Total Hip Arthroplasty https://clinicaltrialsgov/ct2/show/NCT04295408. 2020. PubMed PMID: CN-02083034.
44. Kalashetty M, Khanam U. Comparison of two different types of ultrasound guided nerve blocks for pain relief after hip surgery https://trialsearchwhoint/Trial2aspx?TrialID=CTRI/2023/11/059466. 2023. PubMed PMID: CN-02626009.
45. Kang Z. Comparison of analgesia between PENG plus LFCN block and supra-inguinal fascia iliaca block after hip and femur surgeries https://trialsearchwhoint/Trial2aspx?TrialID=ChiCTR2200065793. 2022. PubMed PMID: CN-02569587.
46. Khandelwal M, Darshan N. Comparison of pericapsular nerve group block and femoral nerve block for analgesia after hip fracture surgery https://trialsearchwhoint/Trial2aspx?TrialID=CTRI/2023/06/053932. 2023. PubMed PMID: CN-02576800.
47. Kukreja P. Trial Comparing Impact of PENG Block on Quality of Recovery Compared to No-block for Primary Total Hip Arthroplasty https://clinicaltrialsgov/ct2/show/NCT04591353. 2020. PubMed PMID: CN-02196581.
48. Kumar A. Study to compare postoperative analgesic efficacy between Fascia Iliaca block and PENG block https://trialsearchwhoint/Trial2aspx?TrialID=CTRI/2020/02/023640. 2020. PubMed PMID: CN-02167172.
49. Kumar A, Raushan R. efficacy of iliopsoas plane block vs pericapsular nerve block in hip replacement surgery https://trialsearchwhoint/Trial2aspx?TrialID=CTRI/2024/02/062713. 2024. PubMed PMID: CN-02674651.
50. Lai GY. Study on the effective volume of pericapsular nerve group block in hip surgery https://trialsearchwhoint/Trial2aspx?TrialID=ChiCTR2200055345. 2022. PubMed PMID: CN-02525037.
51. Lee JK, Hwang DS. A comparison of the analgesic efficacy of pericapsular nerve group blocking vs periarticular injection after total hip arthroplaslty - Prospective study http://wwwwhoint/trialsearch/Trial2aspx?TrialID=KCT0006049. 2021. PubMed PMID: CN-02448872.
52. Li XP, Wang F. Comparison of ultrasound-guided continuous pericapsular nerve group block and continuous fascia iliaca compartment block for hip replacement surgery in elderly patients: a single-center,prospective,randomized controlled trial https://trialsearchwhoint/Trial2aspx?TrialID=ChiCTR2100046433. 2021. PubMed PMID: CN-02376527.
53. Li ZY, Shu LP. Study on the analgesic effect of pericapsular nerve group(PENG) block methods of bupivacaine liposome after total hip arthroplasty https://trialsearchwhoint/Trial2aspx?TrialID=ChiCTR2300076780. 2023. PubMed PMID: CN-02652514.
54. LifeBridge Health. The Pericapsular Nerve Block in Total Hip Arthroplasty https://clinicaltrialsgov/show/NCT04729686. 2020. PubMed PMID: CN-02234539.
55. Lin DY. A Randomised Control Trial comparing the pain relief effect of the pericapsular nerve group (PENG) block to pretend 'sham' blocks in hip replacement surgery https://trialsearchwhoint/Trial2aspx?TrialID=ACTRN12621000068864. 2021. PubMed PMID: CN-02239606.
56. Lin DY. PENG vs placebo 'sham' block RCT https://trialsearchwhoint/Trial2aspx?TrialID=NL9044. 2020. PubMed PMID: CN-02239106.
57. Lin DY. PENG vs placebo 'sham' block RCT https://trialsearchwhoint/Trial2aspx?TrialID=NL9147. 2020. PubMed PMID: CN-02240686.
58. Lin DY. Pericapsular nerve group block versus intrathecal morphine in hip arthroplasty: a non-blinded randomised controlled trial https://trialsearchwhoint/Trial2aspx?TrialID=NL8952. 2020. PubMed PMID: CN-02189073.
59. Lin DY. Pericapsular nerve group block versus intrathecal morphine in hip arthroplasty: a non-blinded randomised controlled trial https://trialsearchwhoint/Trial2aspx?TrialID=NL8952. 2020. PubMed PMID: CN-02189073.
60. Lin DY. Randomized Control Trial comparing PENG (PEricapsular Nerve Group) block to femoral nerve block in patients with a neck of femur fracture https://trialsearchwhoint/Trial2aspx?TrialID=ACTRN12620000298910. 2020. PubMed PMID: CN-02165173.
61. Meierhernich R. The Analgesic Efficacy of Pericapsular Nerve Group (PENG) Block in Patients Undergoing Primary Total Hip Arthoplasty https://clinicaltrialsgov/ct2/show/NCT06557044. 2024. PubMed PMID: CN-02739330.
62. Mitra S. Comparison of pain relief between ultrasound guided continuous pericapsular nerve group block and lumbar plexus block after undergoing surgery for unilateral hip fracture https://trialsearchwhoint/Trial2aspx?TrialID=CTRI/2021/07/034895. 2021. PubMed PMID: CN-02328089.
63. Morales-Avalos R. Ropivacaine and Midazolam by Intraarticular vs Epidural Administration in Arthroscopic ACL https://clinicaltrialsgov/show/NCT05078372. 2021. PubMed PMID: CN-02332262.
64. Mostafa TAH. Comparison of Ultrasound Guided Caudal Block and Ultrasound Guided Pericapsular Nerve Group Block for Pediatric Hip Surgery https://clinicaltrialsgov/ct2/show/NCT04336085. 2020. PubMed PMID: CN-02091420.
65. Mulier J. Impact of Opioid Free Anesthesia on Outcome After Hip Arthroplasty by Direct Anterior Approach https://clinicaltrialsgov/show/NCT03663426. 2018. PubMed PMID: CN-01663087.
66. National University of Malaysia. Comparing The Effectiveness Of Pericapsular Nerve Group (PENG) Block Versus Supra-Inguinal Fascia Iliaca Compartment Block(S-FICB) In Reducing Positional Pain During Neuraxial Anaesthesia In Hip Fractures Patients https://clinicaltrialsgov/show/NCT05721924. 2023. PubMed PMID: CN-02522894.
67. Nuthep L. Adding pericapsular nerve group (PENG) block for postoperative pain relief following elderly hip fracture surgery: a prospective randomized controlled trial https://trialsearchwhoint/Trial2aspx?TrialID=TCTR20220622003. 2022. PubMed PMID: CN-02430287.
68. Panjiar P. COMPARING THE EASE OF POSITIONING IN ELDERLY PATIENTS WITH HIP FRACTURE USING TWO LOWER LIMB NERVE BLOCKS https://trialsearchwhoint/Trial2aspx?TrialID=CTRI/2023/04/051879. 2023. PubMed PMID: CN-02554624.
69. Park SY, Eom DW. A study on the reduction of total consumption of postoperative opioid after pericapsular nerve group (PENG) blockage in hip surgery http://wwwwhoint/trialsearch/Trial2aspx?TrialID=KCT0006348. 2021. PubMed PMID: CN-02438159.
70. Pattnaik S, Sahoo R. A study to compare ultrasound guided pericapsular nerve group block (PENG) & femoral nerve block for minimizing positional pain during spinal and epidural anaesthesia in hip fractures posted for surgery https://trialsearchwhoint/Trial2aspx?TrialID=CTRI/2024/01/061467. 2024. PubMed PMID: CN-02655806.
71. Poznan University of Medical Sciences. PENG Block for Total Hip Arthroplasty https://clinicaltrialsgov/ct2/show/NCT05944380. 2023. PubMed PMID: CN-02580227.
72. Region Skane. Comparison Between Blocks or Not in Joint Arthroplasty https://clinicaltrialsgov/ct2/show/NCT06230081. 2024. PubMed PMID: CN-02677011.
73. Ruan XC. Sonography-guided Pericapsular Never Group Block for Hip Arthroplasty https://clinicaltrialsgov/show/NCT04480320. 2020. PubMed PMID: CN-02137319.
74. Sahoo S. Pain management of Ultrasound- guided Pericapsular nerve group block in hip Surgeries, A Experimental study https://trialsearchwhoint/Trial2aspx?TrialID=CTRI/2022/11/047699. 2022. PubMed PMID: CN-02512365.
75. Samadpour H. Evaluation of the effect of pre-capsular nerve group block on opoid intra-operative consumption and analgesic effects after hip fracture surgery https://trialsearchwhoint/Trial2aspx?TrialID=IRCT20200314046763N1. 2020. PubMed PMID: CN-02171560.
76. Sameh Abdelkhalik Ahmed Ismaiel, Tanta University. Ultrasound-guided PENG Block in Total Hip Replacement https://clinicaltrialsgov/show/NCT04984109. 2021. PubMed PMID: CN-02297167.
77. Satici MH. Effect of Pericapsular Nerve Block on the Quality of Recovery After Shoulder Arthroscopy https://clinicaltrialsgov/ct2/show/NCT06225089. 2023. PubMed PMID: CN-02679887.
78. Seraglio P. Pericapsular Nerve Group Block for Positional Pain and Postoperative Analgesia in Hip Fractures https://clinicaltrialsgov/show/NCT05079087. 2021. PubMed PMID: CN-02340966.
79. Sidhu GK. Quality ofrecovery with pericapsular nerve block with use of dexamethasone as an adjuvant https://trialsearchwhoint/Trial2aspx?TrialID=CTRI/2020/11/029303. 2020. PubMed PMID: CN-02239052.
80. Singapore General Hospital. Patients With Acute Hip Fractures Will Receive Either the PENG Block or no Block Respectively https://clinicaltrialsgov/show/NCT04996979. 2021. PubMed PMID: CN-02291313.
81. Sonawane K. Analgesic efficacy of Pre-Emptive USG guided Pericapsular Nerve Group (PENG) block vs Femoral nerve block vs articular branch block while positioning the patient during neuraxial anaesthesia for unilateral hip fracture surgery https://trialsearchwhoint/Trial2aspx?TrialID=CTRI/2019/04/018654. 2019. PubMed PMID: CN-01974563.
82. Sujarittham W. The efficacy of ultrasound-guided pericapsular nerve group (PENG) block for postoperative pain control in hip surgery : a double-blind randomised controlled trial https://trialsearchwhoint/Trial2aspx?TrialID=TCTR20190721001. 2019. PubMed PMID: CN-02071757.
83. Tah A, Agrawal N. â??Comparative evaluation of 2 Nerve Blocks (Ultrasound Guided Pericapsular Nerve Group Block with Femoral Nerve Block) for reducing pain associated with positioning for subarachnoid block in patients undergoing hip fracture surgeryâ?? https://trialsearchwhoint/Trial2aspx?TrialID=CTRI/2021/08/035976. 2021. PubMed PMID: CN-02328696.
84. Tsai TY. PENG Block for Traumatic Hip Fracture in the Emergency Department https://clinicaltrialsgov/show/NCT04997785. 2021. PubMed PMID: CN-02297464.
85. Uludag E. GENERAL vs REGIONAL ANESTHESIA ON SLEEP QUALITY FOR HIP ARTROPLASTY PATIENTS https://clinicaltrialsgov/ct2/show/NCT06041711. 2023. PubMed PMID: CN-02599674.
86. University Hospital, Toulouse. Analgesic Effectiveness of PENG Block in Programmed Hip Arthroplasty Surgery https://clinicaltrialsgov/ct2/show/NCT04650100. 2020. PubMed PMID: CN-02206041.
87. University Hospital, Toulouse. Analgesic Effectiveness of PENG Block in Programmed Hip Arthroplasty Surgery https://clinicaltrialsgov/show/NCT04650100. 2020. PubMed PMID: CN-02206041.
88. Vardhan AV. Ultrasound guided Pericapsular Nerve Group block versus Supra Inguinal Approach of Fascia Iliaca block for positioning and postoperative analgesia in patients posted for Hip Surgeries https://trialsearchwhoint/Trial2aspx?TrialID=CTRI/2024/07/069880. 2024. PubMed PMID: CN-02731917.
89. Vested M. Investigating the Effect of Pericapsular Nerve Group (PENG) Block on Postoperative Pain After Peri-acetabular Osteotomy https://clinicaltrialsgov/ct2/show/NCT06406010. 2024. PubMed PMID: CN-02697253.
90. Wake Forest University Health Sciences. PENG Block Versus LP Block for THA Postop Pain https://clinicaltrialsgov/ct2/show/NCT05261009. 2022. PubMed PMID: CN-02382051.
91. Wang J. Analgesic effects of Liposomal bupivacaine vs Bupivacaine Hydrochloride for PENG block in patients undergoing hip fracture surgery https://trialsearchwhoint/Trial2aspx?TrialID=ChiCTR2300077758. 2023. PubMed PMID: CN-02652912.
92. Wang F, Xu HJ. A comparative study of pericapsular nerve group block and quadratus lumborum block in total hip arthroplasty https://trialsearchwhoint/Trial2aspx?TrialID=ChiCTR2100046457. 2021. PubMed PMID: CN-02439500.
93. Yonsei University. Pericapsular Nerve Group (PENG) Block Combined With Periarticular Multimodal Drug Injection (PMDI) Versus Isolated PMDI for Pain Management After Total Hip Arthroplasty: a Randomized Controlled Trial https://clinicaltrialsgov/show/NCT05320913. 2022. PubMed PMID: CN-02388231.
94. Yörükoğlu HU. The Effect of Nociception Level (NOL) Monitoring on Intraoperative Opioid Consumption in Hip Surgeries https://clinicaltrialsgov/ct2/show/NCT06558149. 2024. PubMed PMID: CN-02739355.
95. Zheng XC, Tu WS. Observation of preoperative analgesic effect of bupivacaine liposomes on the pericapsular nerve group block without opioid drugs in elderly patients with hip fractures: a single center, prospective, randomized controlled Study https://trialsearchwhoint/Trial2aspx?TrialID=ChiCTR2400083591. 2024. PubMed PMID: CN-02696947.

**1.2 Conference abstracts (n=34)**

| **No.** | **Author and year** | **Reasons of exclusion** |
| --- | --- | --- |
| 1 | A et al., 2022 | Conference Abstract |
| 2 | Akgul et al., 2023 | Conference Abstract |
| 3 | Al-Ani et al., 2023 | Conference Abstract |
| 4 | Aslan et al., 2023 | Conference Abstract |
| 5 | Azpiazu et al., 2019 | Conference Abstract |
| 6 | Bustillo et al., 2023 | Conference Abstract |
| 7 | Carella et al., 2023 | Conference Abstract |
| 8 | Cesur et al., 2022 | Conference Abstract |
| 9 | Cheong et al., 2023 | Conference Abstract |
| 10 | Chiang et al., 2023 | Conference Abstract |
| 11 | Daun et al., 2023 | Conference Abstract |
| 12 | Debabi et al., 2023 | Conference Abstract |
| 13 | Delgado et al., 2019 | Conference Abstract |
| 14 | Dusak et al., 2023 | Conference Abstract |
| 15 | Garcia et al., 2021 | Conference Abstract |
| 16 | Gargano et al., 2021 | Conference Abstract |
| 17 | Gonçalves et al., 2022 | Conference Abstract |
| 18 | Headon et al., 2023 | Conference Abstract |
| 19 | Jin et al., 2023 | Conference Abstract |
| 20 | Jung 2023 | Conference Abstract |
| 21 | Kurian et al., 2019 | Conference Abstract |
| 22 | McDonald et al., 2021 | Conference Abstract |
| 23 | McEwan et al., 2023 | Conference Abstract |
| 24 | Narayanan et al., 2021 | Conference Abstract |
| 25 | Neji et al., 2023 | Conference Abstract |
| 26 | Polmear et al., 2020 | Conference Abstract |
| 27 | Pourkashanian et al., 2019 | Conference Abstract |
| 28 | Roriz et al., 2019 | Conference Abstract |
| 29 | Sahu et al., 2023 | Conference Abstract |
| 30 | Silva et al., 2019 | Conference Abstract |
| 31 | Soares et al., 2021 | Conference Abstract |
| 32 | Soares et al., 2019 | Conference Abstract |
| 33 | Teston et al., 2023 | Conference Abstract |
| 34 | Yörükoǧlu et al., 2022 | Conference Abstract |

**References**

1. A OM, Deli M, B OD, Shorten G. A comparison of hip flexor motor function and analgesia associated with pericapsular nerve group blockade and femoral nerve blockade for patients undergoing neck of femur fracture surgery. Anaesthesia. 2022;77(SUPPL 2):51. doi: 10.1111/anae.15631. PubMed PMID: CN-02364271.
2. Akgul IA, Canbolat N, Buget MI, Altun D, Sen C, Koltka K. COMPARISON OF LOCAL ANAESTHETIC CONCENTRATION IN PERICAPSULAR NERVE GROUP (PENG) BLOCK FOR TOTAL HIP ARTHROPLASTY: a PROSPECTIVE RANDOMIZED DOUBLE-BLIND CONTROLLED TRIAL. Regional anesthesia and pain medicine. 2023;48:A26‐A7. doi: 10.1136/rapm-2023-ESRA.44. PubMed PMID: CN-02698586.
3. Al-Ani T, Inglis L. PERICAPSULAR NERVE GROUP BLOCK ADDED TO FEMORAL AND LATERAL FEMORAL CUTANEOUS NERVE BLOCK USED FOR POSITIONING PATIENTS WITH HIP FRACTURES FOR SPINAL ANAESTHESIA. Regional Anesthesia and Pain Medicine. 2023;48:A328. doi: 10.1136/rapm-2023-ESRA.624.
4. Aslan M, Kilicaslan A, Funda G. RANDOMISED COMPARISON BETWEEN PERICAPSULAR NERVE GROUP BLOCK WITH LATERAL FEMORAL CUTANEOUS NERVE BLOCK AND QUADRATUS LUMBORUM BLOCK FOR POSTOPERATIVE ANALGESIA IN HIP SURGERY. Regional anesthesia and pain medicine. 2023;48:A43. doi: 10.1136/rapm-2023-ESRA.71. PubMed PMID: CN-02698617.
5. Azpiazu N, Perez P, Taibo JM, Ortega U, Diez E, Velasco C. Pericapsular nerve group (PENG) block after total hip arthroplasty a report of five cases. Regional Anesthesia and Pain Medicine. 2019;44(10):A200. doi: 10.1136/rapm-2019-ESRAABS2019.340.
6. Bustillo RG, De Miguel Manso S, Pastor CG, Quirós BS. PERICAPSULAR BLOCK FOR ANALGESIA IN SURGERY OF THE LOWER EXTREMITY. Regional Anesthesia and Pain Medicine. 2023;48:A287. doi: 10.1136/rapm-2023-ESRA.545.
7. Carella M, Beck F, Piette N, Lecoq JP, Bonhomme V. COMPARISON BETWEEN PERIARTICULAR INFILTRATION, PERICAPSULAR NERVE GROUP AND SUPRAINGUINAL FASCIA ILIACA BLOCKS ON POSTOPERATIVE FUNCTIONAL RECOVERY IN TOTAL HIP ARTHROPLASTY: PRELIMINARY RESULTS FROM A RANDOMIZED CONTROLLED CLINICAL STUDY. Regional anesthesia and pain medicine. 2023;48:A24‐A5. doi: 10.1136/rapm-2023-ESRA.42. PubMed PMID: CN-02698579.
8. Cesur S, Yörükoǧlu HU, Aksu C, Kuş A. ANALGESIA FOR HIP SURGERY WITH CONTINUOUS PENG BLOCK IN A FRAIL PATIENT. Regional Anesthesia and Pain Medicine. 2022;47:A192. doi: 10.1136/rapm-2022-ESRA.300.
9. Cheong KM, Tsai TY. Comparison Between Femoral Nerve Block and Pericapsular Nerve Group Block in Extracapsular Hip Fractures. Academic Emergency Medicine. 2023;30:222-3. doi: 10.1111/acem.14718.
10. Chiang CH, Tsai TY, Chau SW, Chu SE, Sun JT. A Comparison of Pericapsular Nerve Group Block and Intravenous Morphine in Patients With Hip Fracture. Academic emergency medicine. 2023;30:223. doi: 10.1111/acem.14718. PubMed PMID: CN-02575392.
11. Daun J, Ha-Jung K, Yeon Ju K, Ji-In P, Uk KW, Hyungtae K, et al. COMPARISON OF PERICAPSULAR NERVE GROUP (PENG) BLOCK WITH SUPRAINGUINAL FASCIA ILIACA COMPARTMENT BLOCK (FICB) ON DYNAMIC PAIN IN PATIENTS WITH HIP FRACTURES: a PROSPECTIVE RANDOMIZED CONTROLLED TRIAL. Regional anesthesia and pain medicine. 2023;48:A105‐A6. doi: 10.1136/rapm-2023-ESRA.185. PubMed PMID: CN-02698631.
12. Debabi C, Yedes A, Njima OB, Methneni M, Ketata H, Mazlout M, et al. QUALITY OF RECOVERY AFTER HIP FRACTURE SURGERY: PERICAPSULAR NERVE GROUP BLOCK VERSUS FASCIA ILIACA COMPARTMENT BLOCK. Regional anesthesia and pain medicine. 2023;48:A23‐A4. doi: 10.1136/rapm-2023-ESRA.40. PubMed PMID: CN-02698573.
13. Delgado A, Portugalyan M. Pericapsular nerve group block (PENG) for postoperative analgesia following femoral neck fracture surgery (dynamic hip screw). Regional Anesthesia and Pain Medicine. 2019;44(10):A221-A2. doi: 10.1136/rapm-2019-ESRAABS2019.388.
14. Dusak IWS, Ciatawi K. The Use of Pericapsular Nerve Group (PENG) Block in Hip Surgery. Orthopaedic Journal of Sports Medicine. 2023;11(2). doi: 10.1177/2325967121s00879.
15. Garcia H, Moreno N, Quintero O, Vallejo J. Pericapsular nerve group block in total hip replacement surgery: A case series description. Anesthesia and Analgesia. 2021;133(3 SUPPL 2):1699.
16. Gargano F, Strumia A, Costa F, Pascarella G, Rizzo S, Antinolfi V, et al. Peripheral nerve blocks allow hip fracture surgical management in an extremely fragile patient: No peng no game. Regional Anesthesia and Pain Medicine. 2021;70(SUPPL 1):A91. doi: 10.1136/rapm-2021-ESRA.173.
17. Gonçalves D, Peixoto De Sousa C, Teixeira F, Morais D, Santos C, Norte G. PENG BLOCK FOR POSTOPERATIVE ANALGESIA IN PATIENTS UNDERGOING SURGICAL CORRECTION OF TRAUMATIC PROXIMAL FEMORAL FRACTURES OR TOTAL HIP ARTHROPLASTY. Regional Anesthesia and Pain Medicine. 2022;47:A255. doi: 10.1136/rapm-2022-ESRA.432.
18. Headon H, Yoon S, McKavanagh E, Van Ross J, Uzkalniene V, Edipoglu I. ACHIEVING 'PENG' HIP FLEXION FOLLOWING TOTAL HIP ARTHROPLASTY: A COMPARISON BETWEEN THE PENG AND FASCIA ILIACA BLOCKS IN TOTAL HIP ARTHROPLASTY. Regional Anesthesia and Pain Medicine. 2023;48:A124. doi: 10.1136/rapm-2023-ESRA.216.
19. Jin Z, Sugiyama D, Higo F, Hirata T, Kobayashi O, Ueda K. :THE EFFICACY OF PERICAPSULAR NERVE GROUP (PENG) BLOCK IN PREOPERATIVE REHABILITATION FOR PATIENTS WITH FEMORAL-NECK FRACTURES: A PILOT STUDY. Regional Anesthesia and Pain Medicine. 2023;48:A60. doi: 10.1136/rapm-2023-ESRA.100.
20. Jung Y. EFFECT AND METHOD OF CONTINUOUS PERICAPSULAR NERVE GROUP BLOCK IN FEMUR FRACTURE PATIENTS UNDERGOING TOTAL HIP ARTHROPLASTY: CASE REPORT. Regional Anesthesia and Pain Medicine. 2023;48:A312-A3. doi: 10.1136/rapm-2023-ESRA.595.
21. Kurian J, George S, Babu I. Analgesia for proximal hip fracture with pericapsular nerve group (PENG) block-a case report. Regional Anesthesia and Pain Medicine. 2019;44(10):A147. doi: 10.1136/rapm-2019-ESRAABS2019.211.
22. McDonald D, Tilak D. Suprainguinal fascia iliaca with pericapsular nerve group block for fractured neck of femur vs. traditional approaches: A better way? Anaesthesia. 2021;76(SUPPL 2):138. doi: 10.1111/anae.15338.
23. McEwan A, Ramachandran A, Manyapu M, Singh M, Halperin M, Montenegro MA, et al. Novel PENG Block SIM Training for Hip Fractures in the Emergency Department. Journal of Emergency Medicine. 2023;64(3):416-7. doi: 10.1016/j.jemermed.2023.03.011.
24. Narayanan S, Bojaraaj K, Murugesan B. Role of PENG Block In Hip Surgeries: our Experiences in Rural India. Anesthesia and analgesia. 2021;133(3 SUPPL 2):1740‐1. PubMed PMID: CN-02323133.
25. Neji I, Nasri O, Ferchichi S, Messaoud CB, Khiari H, Raddaoui K, et al. EFFICACY OF PERICAPSULAR NERVE GROUP BLOCK AFTER TOTAL HIP ARTHROPLASTY SURGERY. Regional anesthesia and pain medicine. 2023;48:A120‐A1. doi: 10.1136/rapm-2023-ESRA.211. PubMed PMID: CN-02698568.
26. Polmear M, Christensen D, Salfiti C, Wolff A, Scanaliato J. Lumbar plexus block versus peri-capsular injection for hip arthroscopy: a single-blinded randomized controlled trial. Orthopaedic journal of sports medicine. 2020;8(7 SUPPL 6). doi: 10.1177/2325967120s00350. PubMed PMID: CN-02230697.
27. Pourkashanian A, Narayanan M, Dhar M, Padman D. Finding the perfect combination: Pericapsular nerve group (PENG) block utilisation in surgical repair of hip fractures. Regional Anesthesia and Pain Medicine. 2019;44(10):A222. doi: 10.1136/rapm-2019-ESRAABS2019.389.
28. Roriz D, Brandão J, Ribas D, Sá M, Cardoso JM, Caramelo S, et al. Peng block as an analgesic tool for total hip arthroplasty: A case series description. Regional Anesthesia and Pain Medicine. 2019;44(10):A204-A5. doi: 10.1136/rapm-2019-ESRAABS2019.349.
29. Sahu A, Sinha T, Bhoi S, Aggarwal P. 29 Pericapsular Nerve Group Block (PENG) Versus Fascia Iliaca Compartment Block (FICB) for Hip and Femur Fractures in the Emergency Department: a Propensity Score Matched Cohort Study. Annals of emergency medicine. 2023;82(4):S12. doi: 10.1016/j.annemergmed.2023.08.048. PubMed PMID: CN-02612833.
30. Silva R, Almeida P, Veiga J, Castro J, Lakhani R, Ribeiro L, et al. Hip arthroscopy-is the pericapsular nerve group block approach plus a subpectineal injection the key to successful analgesia? two case reports. Regional Anesthesia and Pain Medicine. 2019;44(10):A198. doi: 10.1136/rapm-2019-ESRAABS2019.336.
31. Soares J, Noronha D, Veiga M. Efficacy of Continuous Pericapsular Nerve Group (PENG) Block for Pain Relief after Hemiarthroplasty of the Hip: Three Case Reports. Anesthesia and Analgesia. 2021;133(3 SUPPL 2):1780.
32. Soares J, Veiga M, Galacho J, Ormonde L. Efficacy of continuous pericapsular nerve group (PENG) block for pain relief after hemiarthroplasty of the hip: A case report. Regional Anesthesia and Pain Medicine. 2019;44(10):A210. doi: 10.1136/rapm-2019-ESRAABS2019.363.
33. Teston AR, Forteza-Rey NF, Pablo SP, Gonzalez EP, Ricardo CV, Violeta PM, et al. QUALITY OF RECOVERY AFTER PERICAPSULAR NERVE GROUP (PENG) BLOCK FOR PRIMARY TOTAL HIP ARTHROPLASTY UNDER SPINAL ANAESTHESIA. Regional anesthesia and pain medicine. 2023;48:A279. doi: 10.1136/rapm-2023-ESRA.529. PubMed PMID: CN-02698582.
34. Yörükoǧlu HU, Cesur S, Aksu C, Kuş A. OPIOID SPARING EFFECT OF PENG BLOCK IN OPEN REDUCTION OF PEDIATRIC DEVELOPMENTAL DYSPLASIA OF THE HIP: A CASE SERIES. Regional Anesthesia and Pain Medicine. 2022;47:A288. doi: 10.1136/rapm-2022-ESRA.508.

**1.3 Reviews (n=23)**

| **No.** | **Author and year** | **Reasons of exclusion** |
| --- | --- | --- |
| 1 | Amin et al., 2023 | Review |
| 2 | Andrade et al., 2023 | Review |
| 3 | Del Buono et al., 2021 | Review |
| 4 | Farag et al., 2023 | Review |
| 5 | Ferre et al., 2021 | Review |
| 6 | González-Roldán et al., 2020 | Review |
| 7 | Hayashi et al., 2024 | Review |
| 8 | Kim et al., 2023 | Review |
| 9 | Kukreja et al., 2021 | Review |
| 10 | Li et al., 2023 | Review |
| 11 | Mai et al., 2024 | Review |
| 12 | Meints et al., 2019 | Review |
| 13 | Mou et al., 2024 | Review |
| 14 | O'Connor et al., 2024 | Review |
| 15 | Pai et al., 2024 | Review |
| 16 | Peng et al., 2023 | Review |
| 17 | Prakash et al., 2023 | Review |
| 18 | She et al., 2024 | Review |
| 19 | Tanguilig et al., 2024 | Review |
| 20 | Wang et al., 2023 | Review |
| 21 | Ying et al., 2023 | Review |
| 22 | Yu et al., 2023 | Review |
| 23 | Zheng et al., 2023 | Review |

**References**

1. Amin SR, Ahmed F. Comparison between pericapsular nerve group block and fascia iliaca compartment block for perioperative pain control in hip surgeries: A meta-analysis from randomized controlled trials. Egyptian Journal of Anaesthesia. 2023;39(1):185-96. doi: 10.1080/11101849.2023.2180540. PubMed PMID: WOS:000935840400001.
2. Andrade PP, Lombardi RA, Marques IR, Braga ACDNAE, Isaias BRS, Heiser NE. Pericapsular Nerve Group (PENG) block versus fascia iliaca compartment (FI) block for hip surgery: a systematic review and meta-analysis of randomized controlled trials. Brazilian Journal of Anesthesiology (English Edition). 2023;73(6):794-809. doi: 10.1016/j.bjane.2023.07.007.
3. Del Buono R, Padua E, Pascarella G, Costa F, Tognù A, Terranova G, et al. Pericapsular nerve group block: An overview. Minerva Anestesiologica. 2021;87(4):458-66. doi: 10.23736/s0375-9393.20.14798-9.
4. Farag A, Hendi NI, Diab RA. Does pericapsular nerve group block have limited analgesia at the initial post-operative period? Systematic review and meta-analysis. Journal of Anesthesia. 2023;37(1):138-53. doi: 10.1007/s00540-022-03129-5. PubMed PMID: WOS:000879660500001.
5. Ferre F, Menut R, Martin C, Minville V. Perioperative management for total hip arthroplasty. Anesthesie & Reanimation. 2021;7(4):279-86. doi: 10.1016/j.anrea.2021.04.002. PubMed PMID: WOS:000674700500008.
6. González-Roldán AM, Terrasa JL, Sitges C, van der Meulen M, Anton F, Montoya P. Age-Related Changes in Pain Perception Are Associated With Altered Functional Connectivity During Resting State. Front Aging Neurosci. 2020;12:116. doi: 10.3389/fnagi.2020.00116. PubMed PMID: 32457594; PubMed Central PMCID: PMCQ2.
7. Hayashi M, Yamamoto N, Kuroda N, Kano K, Miura T, Kamimura Y, et al. Peripheral Nerve Blocks in the Preoperative Management of Hip Fractures: A Systematic Review and Network Meta-Analysis. Annals of Emergency Medicine. 2024;83(6):522-38. doi: 10.1016/j.annemergmed.2024.01.024. PubMed PMID: WOS:001244217300001.
8. Kim E, Shin WC, Lee SM, Choi MJ, Moon NH. Efficacy of Pericapsular Nerve Group Block for Pain Reduction and Opioid Consumption after Total Hip Arthroplasty: A Meta-Analysis of Randomized Controlled Trials. Hip & pelvis. 2023;35(2):63-72. doi: 10.5371/hp.2023.35.2.63. PubMed PMID: MEDLINE:37323546.
9. Kukreja P, Mason L, Feinstein J, Morris SE, Kalagara H. Regional Anesthesia for Total Hip Arthroplasty: Essential Anatomy, Techniques, and Current Literature Review. Current Anesthesiology Reports. 2021;11(4):457-66. doi: 10.1007/s40140-021-00487-w. PubMed PMID: WOS:000701720500001.
10. Li C, Liu Z, Chen M, Song C, Ma X, Zhou X. Comparison of pericapsular nerve group block and fascia iliaca compartment block for analgesia following hip fracture surgery: a meta-analysis. Chinese Journal of Anesthesiology. 2023;43(1):56-61. doi: 10.3760/cma.j.cn131073.20221211.00112.
11. Mai M, van Stralen RA, Moerman S, van Bergen CJA. Postoperative Cast Immobilization Might Be Unnecessary after Pelvic Osteotomy for Children with Developmental Hip Dysplasia: A Systematic Review. Surgical Techniques Development. 2024;13(1):9-21. doi: 10.3390/std13010002. PubMed PMID: WOS:001192823800001.
12. Meints SM, Cortes A, Morais CA, Edwards RR. Racial and ethnic differences in the experience and treatment of noncancer pain. Pain Manag. 2019;9(3):317-34. doi: 10.2217/pmt-2018-0030. PubMed PMID: 31140916.
13. Mou Z, Xiang L, Ni Y. Comparative effectiveness of pericapsular nerve group block versus fascia iliac compartment block on postoperative wound pain management in patients undergoing hip fracture surgery: A systematic review and meta-analysis. International Wound Journal. 2024;21(2). doi: 10.1111/iwj.14637. PubMed PMID: WOS:001159070000001.
14. O'Connor KP, Davidson JC, Nepple JJ, Clohisy JC, Willey MC. Pain Management for Periacetabular Osteotomy: A Systematic Review. The Iowa orthopaedic journal. 2024;44(1):125-32. PubMed PMID: MEDLINE:38919337.
15. Pai P, Amor D, Lai YH, Echevarria GC. Use and Clinical Relevancy of Pericapsular Nerve Block (PENG) in Total Hip Arthroplasty: A Systematic Review and Meta-analysis. Clinical Journal of Pain. 2024;40(5):320-32. doi: 10.1097/ajp.0000000000001196.
16. Peng S, Zhang M, Jin J, MacCormick AD. The effectiveness of venous thromboembolism prophylaxis interventions in trauma patients: A systematic review and network meta-analysis. Injury. 2023;54(12):111078. Epub 20231005. doi: 10.1016/j.injury.2023.111078. PubMed PMID: 37865011.
17. Prakash J, Rochwerg B, Saran K, Yadav AK, Bhattacharya PK, Kumar A, et al. Comparison of analgesic effects of pericapsular nerve group block and fascia iliaca compartment block during hip arthroplasty: A systematic review and meta.analysis of randomised controlled trials. Indian Journal of Anaesthesia. 2023;67(11):962-72. doi: 10.4103/ija.ija_672_23.
18. She C, Liu H. The efficacy of pericapsular nerve group block for reducing pain and opioid consumption after total hip arthroplasty: a systematic review and meta-analysis. Journal of Orthopaedic Surgery and Research. 2024;19(1). doi: 10.1186/s13018-024-04707-x. PubMed PMID: WOS:001198097400001.
19. Tanguilig G, Dhillon J, Scillia AJ, Heard WMR, Kraeutler MJ. The Addition of a Pericapsular Nerve Group Block for Postoperative Pain Control Does Not Result in Less Narcotic Use After Hip Arthroscopy: A Systematic Review. Arthroscopy, Sports Medicine, and Rehabilitation. 2024;6(2). doi: 10.1016/j.asmr.2024.100894.
20. Wang Y, Wen H, Wang M, Lu M. The Efficiency of Ultrasound-Guided Pericapsular Nerve Group Block for Pain Management after Hip Surgery: A Meta-analysis. Pain and Therapy. 2023;12(1):81-92. doi: 10.1007/s40122-022-00463-0. PubMed PMID: WOS:000895639600001.
21. Ying H, Chen L, Yin D, Ye Y, Chen J. Efficacy of pericapsular nerve group block vs. fascia iliaca compartment block for Hip surgeries: A systematic review and meta-analysis. Frontiers in Surgery. 2023;10. doi: 10.3389/fsurg.2023.1054403. PubMed PMID: WOS:000944973500001.
22. Yu L, Shen X, Liu H. The efficacy of pericapsular nerve group block for postoperative analgesia in patients undergoing hip surgery: A systematic review and meta-analysis of randomized controlled trials. Frontiers in Medicine. 2023;10. doi: 10.3389/fmed.2023.1084532. PubMed PMID: WOS:000946533200001.
23. Zheng J, Du L, Chen G, Zhang L, Deng X, Zhang W. Efficacy of pericapsular nerve group (PENG) block on perioperative pain management in elderly patients undergoing hip surgical procedures: a protocol for a systematic review with meta-analysis and trial sequential analysis. Bmj Open. 2023;13(1). doi: 10.1136/bmjopen-2022-065304. PubMed PMID: WOS:001020797800006.

**1.4 Others (n=10)**

| **No.** | **Author and year** | **Reasons of exclusion** |
| --- | --- | --- |
| 1 | Ayisi 2022 | Dissertation/Thesis |
| 2 | de Oliveira Dias 2023 | Dissertation/Thesis |
| 3 | Hua et al., 2022 | Retracted Publication |
| 4 | Kollmorgen 2024 | Editorial Material |
| 5 | Ueshima et al., 2022 | Retracted Publication |
| 6 | Zaragoza-Lemus et al. 2020 | brief-report |
| 7 | Zaragoza-Lemus et al. 2021 | case-report |
| 8 | Zhang 2022 | Editorial Material |
| 9 | Zheng et al., 2022 | Erratum |
| 10 | Zheng et al., 2022 | Erratum |

**References**

1. Ayisi J. Postoperative Pain Management in Hip Preservation Surgeries [Dissertation/Thesis]2022.
2. de Oliveira Dias HJ. Postoperative Acute Pain Management in Hip Arthroplasty: Analgesic Efficacy of Single-Dose Peripheral Nerve Block [Dissertation/Thesis]2023.
3. Hua H, Xu Y, Jiang M, Dai X. Evaluation of Pericapsular Nerve Group (PENG) Block for Analgesic Effect in Elderly Patients with Femoral Neck Fracture Undergoing Hip Arthroplasty. Journal of healthcare engineering. 2022;2022:7452716. doi: 10.1155/2022/7452716. PubMed PMID: CN-02375848.
4. Kollmorgen R. Editorial Commentary: The Addition of the Pericapsular Nerve Group Block Leads to Reduced Pain Up to 24 Hours After Hip Arthroscopy. Arthroscopy-the Journal of Arthroscopic and Related Surgery. 2024;40(2):381-3. doi: 10.1016/j.arthro.2023.07.014. PubMed PMID: WOS:001186055500001.
5. Ueshima H, Otake H. Retraction notice to “SpPericapsular nerve group (PENG) block is effective for dislocation of the hip joint” [J. Clin. Anesth. 52C(2019) 83, (S0952818018310596), (10.1016/j.jclinane.2018.09.022)]. Journal of Clinical Anesthesia. 2022;80. doi: 10.1016/j.jclinane.2022.110835.
6. Zaragoza-Lemus G, Portela-Ortiz JM, Díaz-Guevara G. Bloqueo del grupo de nervios pericapsulares (PENG) para cirugía de cadera. Revista mexicana de anestesiología. 2020;43(1):69-72. doi: 10.35366/cma201m. PubMed PMID: SCIELO:S0484-79032020000100069.
7. Zaragoza-Lemus G, Céspedes-Korrodi MÁ, Hernández-Rodríguez D, Mancera-Rangel M. Colocación de catéter ecoguiado para bloqueo PENG continuo en Cirugía de Cadera. Revista mexicana de anestesiología. 2021;44(3):233-6. PubMed PMID: SCIELO:S0484-79032021000300233.
8. Zhang AL. Editorial Commentary: Quadratus Lumborum Analgesia for Hip Arthroscopy: The New Kid on the (Nerve) Block. Arthroscopy - Journal of Arthroscopic and Related Surgery. 2022;38(3):816-7. doi: 10.1016/j.arthro.2021.08.031.
9. Zheng J, Pan D, Zheng B, Ruan X. Correction: Preoperative pericapsular nerve group (PENG) block for total hip arthroplasty: a randomized, placebo-controlled trial. Regional anesthesia and pain medicine. 2022;47(7):e4-e. doi: 10.1136/rapm-2021-103228corr1. PubMed PMID: MEDLINE:35609891.
10. Zheng J, Pan D, Zheng B. Erratum: Preoperative pericapsular nerve group (PENG) block for total hip arthroplasty: A randomized, placebo-controlled trial(Regional Anesthesia and Pain Medicine (2022) 47 (155–160) DOI: 10.1136/rapm-2021-103228). Regional Anesthesia and Pain Medicine. 2022;47(7):E4. doi: 10.1136/rapm-2021-103228corr1.

**2. Records excluded by titles and abstracts (n=90)**

**2.1 Unrelated topics (n=33)**

| **No.** | **Author and year** | **Reasons of exclusion** |
| --- | --- | --- |
| 1 | Al-Ghadir et al., 2009 | Unrelated topics |
| 2 | Ayisi 2022 | Unrelated topics |
| 3 | Bulut et al., 2021 | Unrelated topics |
| 4 | Correia et al., 2024 | Unrelated topics |
| 5 | Danisman et al., 2023 | Unrelated topics |
| 6 | Ezirmik et al., 2012 | Unrelated topics |
| 7 | Hamra et al., 2023 | Unrelated topics |
| 8 | Huang et al., 2024 | Unrelated topics |
| 9 | Jadon et al., 2020 | Unrelated topics |
| 10 | Jamil et al., 2022 | Unrelated topics |
| 11 | Karaoğlan et al., 2024 | Unrelated topics |
| 12 | Kim et al., 2024 | Unrelated topics |
| 13 | Kitcharanant et al., 2022 | Unrelated topics |
| 14 | Kose et al., 2021 | Unrelated topics |
| 15 | Kose et al., 2023 | Unrelated topics |
| 16 | Lawrenson et al., 2020 | Unrelated topics |
| 17 | Martínez-Álvarez et al., 2011 | Unrelated topics |
| 18 | Mosleh-Shirazi et al., 2022 | Unrelated topics |
| 19 | Nan et al., 2024 | Unrelated topics |
| 20 | Ng et al., 2023 | Unrelated topics |
| 21 | Peng et al., 2011 | Unrelated topics |
| 22 | Polesello et al., 2023 | Unrelated topics |
| 23 | Reza et al., 2024 | Unrelated topics |
| 24 | Salgado-Garcia et al., 2024 | Unrelated topics |
| 25 | Schejbalova et al., 2007 | Unrelated topics |
| 26 | Su et al., 2022 | Unrelated topics |
| 27 | Umeh et al., 2023 | Unrelated topics |
| 28 | Wang et al., 2023 | Unrelated topics |
| 29 | Wang et al., 2022 | Unrelated topics |
| 30 | Wen et al., 2024 | Unrelated topics |
| 31 | Wu et al., 2010 | Unrelated topics |
| 32 | Yilar et al., 2020 | Unrelated topics |
| 33 | Zorer et al., 2002 | Unrelated topics |

**References**

1. Al-Ghadir M, Masquijo JJ, Guerra LA, Willis B. Combined Femoral and Pelvic Osteotomies Versus Femoral Osteotomy Alone in the Treatment of Hip Dysplasia in Children With Cerebral Palsy. Journal of Pediatric Orthopaedics. 2009;29(7):779-83. doi: 10.1097/BPO.0b013e3181b76968. PubMed PMID: WOS:000270499800022.
2. Ayisi J. Postoperative Pain Management in Hip Preservation Surgeries2022.
3. Bulut M, Azboy I, Ozkul E, Karakurt L. Comparison of Iliac and Femoral Autograft Practices in Pemberton Pelvic Osteotomy. Journal of Pediatric Orthopaedics. 2021;41(1):46-50. doi: 10.1097/bpo.0000000000001665. PubMed PMID: WOS:000599710100017.
4. Correia R, Oliveira L, Andrade I, de Castro Correia M, Gonçalves E, Borges A, et al. Ultrasound-Guided Radiofrequency Ablation for Chronic Hip Pain Due to Osteoarthritis. Cureus. 2024;16(2):e53743. Epub 2024/03/11. doi: 10.7759/cureus.53743. PubMed PMID: 38465076; PubMed Central PMCID: PMCPMC10920965.
5. Danisman M, Cetik RM, Tuncay O, Yilmaz G. Intraoperative medial wall disruption in Dega pelvic osteotomy Does it effect the radiographic outcome at medium-term? Saudi Medical Journal. 2023;44(7):687-93. doi: 10.15537/smj.2023.44.7.20230192. PubMed PMID: WOS:001045120900009.
6. Ezirmik N, Yildiz K. Advantages of Single-stage Surgical Treatment with Salter Innominate Osteotomy and Pemberton Pericapsular Osteotomy for Developmental Dysplasia of Both Hips. Journal of International Medical Research. 2012;40(2):748-55. doi: 10.1177/147323001204000240. PubMed PMID: WOS:000304577100040.
7. Hamra P, Sau AS, Junior WR, Rabelo NDDA, Polesello GC. Block of the Pericapsular Nerve Group of the Hip with and without Ultrasound Guidance: Comparative Cadaveric Study. Revista brasileira de ortopedia. 2023;58(4):e646-e52. doi: 10.1055/s-0042-1758367. PubMed PMID: MEDLINE:37663182.
8. Huang Y, Lu Y, Wang J, Lu Q, Bao HF, Liu L, et al. Effect of Pericapsular Nerve Group Block with Different Concentrations and Volumes of Ropivacaine on Functional Recovery in Total Hip Arthroplasty: a Randomized, Observer-Masked, Controlled Trial. Journal of pain research. 2024;17:677‐85. doi: 10.2147/jpr.S445000. PubMed PMID: CN-02665718.
9. Jadon A, Sinha N, Chakraborty S, Singh B, Agrawal A. Pericapsular nerve group (PENG) block: A feasibility study of landmark based technique. Indian Journal of Anaesthesia. 2020;64(8):710-3. doi: 10.4103/ija.IJA_388_20.
10. Jamil K, Saharuddin R, Abd Rasid AF, Abd Rashid AH, Ibrahim S. Outcome of Open Reduction Alone or with Concomitant Bony Procedures for Developmental Dysplasia of the Hip (DDH). Children-Basel. 2022;9(8). doi: 10.3390/children9081213. PubMed PMID: WOS:000846029100001.
11. Karaoğlan M, Küçükçay Karaoğlan B. PENG for chronic pain: the clinical effectiveness of pericapsular nerve group block in chronic hip pain. HIP International. 2024. doi: 10.1177/11207000241227542.
12. Kim JY, Lee JS, Kim JY, Yoon EJ, Lee W, Lee S, et al. Iliopsoas plane block does not improve pain after primary total hip arthroplasty in the presence of multimodal analgesia: a single institution randomized controlled trial. Regional Anesthesia and Pain Medicine. 2024. doi: 10.1136/rapm-2023-105092. PubMed PMID: WOS:001153344400001.
13. Kitcharanant N, Leurcharusmee P, Wangtapun P, Kantakam P, Maikong N, Mahakkanukrauh P, et al. Surgeon-performed pericapsular nerve group (PENG) block for total hip arthroplasty using the direct anterior approach: A cadaveric study. Regional Anesthesia and Pain Medicine. 2022. doi: 10.1136/rapm-2022-103482.
14. Kose M, Yilar S, Topal M, Tuncer K, Aydin A, Zencirli K. Simultaneous versus staged surgeries for the treatment of bilateral developmental hip dysplasia in walking age: A comparison of complications and outcomes. Joint Diseases and Related Surgery. 2021;32(3):605-10. doi: 10.52312/jdrs.2021.38. PubMed PMID: WOS:000721982000007.
15. Kose SG, Kose HC, Celikel F, Tulgar S, Akkaya OT. Ultrasound-guided PENG block versus intraarticular corticosteroid injection in hip osteoarthritis: a randomised controlled study. The Korean journal of pain. 2023;36(2):195‐207. doi: 10.3344/kjp.22325. PubMed PMID: CN-02553511.
16. Lawrenson PR, Vicenzino BT, Hodges PW, Crossley KM, Heerey JJ, Semciw AI. Pericapsular hip muscle activity in people with and without femoroacetabular impingement. A comparison in dynamic tasks. Physical Therapy in Sport. 2020;45:135-44. doi: 10.1016/j.ptsp.2020.06.004.
17. Martínez-Álvarez S, Azorín D, Epeldegui T, Forriol F. Avascular necrosis of the femoral head: Experimental study in lambs. Trauma (Spain). 2011;22(3):188-96.
18. Mosleh-Shirazi A, O’Donnell B. The Analgesic Efficacy of Different Techniques Surrounding Regional Anesthesia of the Lumbar Plexus and its Terminal Branches for Hip Fracture Surgeries. 2022.
19. Nan Y, Wang YY, Jiang XB, Bai Y, Li JL, Luo JY, et al. Efficacy and safety analysis of bupivacaine liposome for pericapsular nerve group block for postoperative analgesia in elderly patients with hip replacement. Zhonghua yi xue za zhi. 2024;104(3):180‐5. doi: 10.3760/cma.j.cn112137-20230810-00190. PubMed PMID: CN-02663008.
20. Ng TKT, Peng P, Chan WS. Posterior hip pericapsular neurolysis (PHPN) for inoperable hip fracture: An adjunct to anterior hip pericapsular neurolysis. Regional Anesthesia and Pain Medicine. 2023. doi: 10.1136/rapm-2021-103023.
21. Peng KT, Kuo LT, Hsu WH, Huang TW, Tsai YH. The effect of endoskeleton on antibiotic impregnated cement spacer for treating deep hip infection. BMC Musculoskeletal Disorders. 2011;12. doi: 10.1186/1471-2474-12-10.
22. Polesello GC, Sau AS, Rudelli M, Junior WR, Rabelo NDDA. Accuracy of Hip Pericapsular Nerve Block (PENG) without Ultrasound Aid in Patients with Hip Pain. Revista brasileira de ortopedia. 2023;58(6):e932-e8. doi: 10.1055/s-0043-1776909. PubMed PMID: MEDLINE:38077758.
23. Reza PC, Vazquez MG, Rodriguez LS, Castro AV, Lopez-Lopez D, Romar AG. Retrospective analysis of regional anaesthesia in hip surgery: A clinical audit. Revista Espanola De Anestesiologia Y Reanimacion. 2024;71(3):160-70. doi: 10.1016/j.redar.2023.07.004. PubMed PMID: WOS:001200251300001.
24. Salgado-Garcia D, Diaz-Alvarez A, Gonzalez-Rodriguez JL, Lopez-Iglesias MR, Sanchez-Lopez E, Sanchez-Ledesma MJ, et al. Comparison of the Analgesic Efficacy between Levobupivacaine 0.25% and Ropivacaine 0.375% for PENG (Pericapsular Nerve Group) Block in the Context of Hip Fracture Surgery of Elderly Patients: a Single-Center, Randomized, and Controlled Clinical Trial. Journal of clinical medicine. 2024;13(3). doi: 10.3390/jcm13030770. PubMed PMID: CN-02673331.
25. Schejbalova A, Chladek P. Acetabuloplasty in cerebral palsy patients. Acta Chirurgiae Orthopaedicae Et Traumatologiae Cechoslovaca. 2007;74(6):382-7. PubMed PMID: WOS:000254257900002.
26. Su Y, Nan G. Modified Pemberton Pelvic Osteotomy Through Inner Ilium Approach for Treatment of Developmental Dysplasia of the Hip in Children. Indian Journal of Orthopaedics. 2022;56(9):1625-33. doi: 10.1007/s43465-022-00676-7. PubMed PMID: WOS:000817054000002.
27. Umeh UO, Kaplan DJ, Diskina D, Commeh E, Cuff G, Hertling A, et al. Transmuscular Quadratus Lumborum Block Does Not Provide Significant Benefit for Primary Hip Arthroscopy with Pericapsular Infiltration: a Randomized Control Trial. Arthroscopy. 2023;39(12):2456‐63. doi: 10.1016/j.arthro.2023.03.025. PubMed PMID: CN-02557735.
28. Wang C-g, Zhang Z-q, Yang Y, Long Y-b, Wang X-l, Ding Y-l. A randomized controlled trial of iliopsoas plane block vs. femoral nerve block for hip arthroplasty. Bmc Anesthesiology. 2023;23(1). doi: 10.1186/s12871-023-02162-5. PubMed PMID: WOS:001004736000003.
29. Wang D, Chen L, Xue J. Comparison of analgesic effect of pericapsular nerve group block of hip joint with different concentrations and volumes of ropivacaine in elderly patients with hip fracture. Journal of Clinical Anesthestology. 2022;38(5):497-502. PubMed PMID: CSCD:7246932.
30. Wen H, Zhang W, Wang Y, Lu M. Effects of Different Volumes of Ropivacaine for Pericapsular Nerve Group Block on Incidence of Quadriceps Weakness and Analgesic Efficacy Following Hip Arthroplasty: a Randomized Controlled Trial. Pain and therapy. 2024;13(3):533‐41. doi: 10.1007/s40122-024-00590-w. PubMed PMID: CN-02673368.
31. Wu K-W, Wang T-M, Huang S-C, Kuo KN, Chen C-W. Analysis of Osteonecrosis Following Pemberton Acetabuloplasty in Developmental Dysplasia of the Hip Long-Term Results. Journal of Bone and Joint Surgery-American Volume. 2010;92A(11):2083-94. doi: 10.2106/jbjs.I.01320. PubMed PMID: WOS:000281908800008.
32. Yilar S, Topal M, Zencirli K, Kose M, Ezirmik N. Comparison of total cost and outcomes between single-stage open reduction and Pemberton periacetabular osteotomy operation and two separate consecutive operations in treatment of bilateral developmental hip dysplasia in children at walking age. Journal of Pediatric Orthopaedics-Part B. 2020;29(3):256-60. doi: 10.1097/bpb.0000000000000714. PubMed PMID: WOS:000528017400007.
33. Zorer G, Bagatur AE. Single-stage bilateral Pemberton's pericapsular osteotomy in bilateral developmental dysplasia of the hip. Acta orthopaedica et traumatologica turcica. 2002;36(4):288‐94. PubMed PMID: CN-00412530.

**2.2 Comparison with other analgesic techniques (n=31)**

| **No.** | **Author and year** | **Reasons of exclusion** |
| --- | --- | --- |
| 1 | Aliste et al., 2021 | Comparison with other analgesic techniques |
| 2 | Bravo et al., 2023 | Comparison with other analgesic techniques |
| 3 | Carella et al., 2023 | Comparison with other analgesic techniques |
| 4 | Choi et al., 2022 | Comparison with other analgesic techniques |
| 5 | Cui et al., 2020 | Comparison with other analgesic techniques |
| 6 | Et et al., 2023 | Comparison with other analgesic techniques |
| 7 | Fontes et al., 2024 | Comparison with other analgesic techniques |
| 8 | Gao et al., 2022 | Comparison with other analgesic techniques |
| 9 | Kadlimatti et al., 2024 | Comparison with other analgesic techniques |
| 10 | Keskes et al., 2023 | Comparison with other analgesic techniques |
| 11 | Lee et al., 2024 | Comparison with other analgesic techniques |
| 12 | Li et al., 2023 | Comparison with other analgesic techniques |
| 13 | Liang et al., 2023 | Comparison with other analgesic techniques |
| 14 | Lin et al., 2021 | Comparison with other analgesic techniques |
| 15 | Liu et al., 2024 | Comparison with other analgesic techniques |
| 16 | Malathi et al., 2024 | Comparison with other analgesic techniques |
| 17 | Mosaffa et al., 2022 | Comparison with other analgesic techniques |
| 18 | Nuthep et al., 2023 | Comparison with other analgesic techniques |
| 19 | Sahoo et al., 2024 | Comparison with other analgesic techniques |
| 20 | Scanaliato et al., 2020 | Comparison with other analgesic techniques |
| 21 | Senthil et al., 2021 | Comparison with other analgesic techniques |
| 22 | Shan et al., 2021 | Comparison with other analgesic techniques |
| 23 | Tang et al., 2023 | Comparison with other analgesic techniques |
| 24 | Vamshi et al., 2023 | Comparison with other analgesic techniques |
| 25 | Wang et al., 2021 | Comparison with other analgesic techniques |
| 26 | Wang et al., 2023 | Comparison with other analgesic techniques |
| 27 | Xie et al., 2020 | Comparison with other analgesic techniques |
| 28 | Ye et al., 2023 | Comparison with other analgesic techniques |
| 29 | Yin et al., 2021 | Comparison with other analgesic techniques |
| 30 | Zheng et al., 2022 | Comparison with other analgesic techniques |
| 31 | Zhou et al., 2023 | Comparison with other analgesic techniques |

**References**

1. Aliste J, Layera S, Bravo D, Jara Á, Muñoz G, Barrientos C, et al. Randomized comparison between pericapsular nerve group (PENG) block and suprainguinal fascia iliaca block for total hip arthroplasty. Regional anesthesia and pain medicine. 2021;46(10):874‐8. doi: 10.1136/rapm-2021-102997. PubMed PMID: CN-02295474.
2. Bravo D, Aliste J, Layera S, Fernandez D, Erpel H, Aguilera G, et al. Randomized clinical trial comparing pericapsular nerve group (PENG) block and periarticular local anesthetic infiltration for total hip arthroplasty. Regional anesthesia and pain medicine. 2023;48(10):489‐94. doi: 10.1136/rapm-2023-104332. PubMed PMID: CN-02526644.
3. Carella M, Beck F, Piette N, Denys S, Lecoq J-P, Bonhomme VLL. Comparison between supra-inguinal fascia iliaca and pericapsular nerve group blocks on postoperative pain and functional recovery after total hip arthroplasty <i>A noninferiority randomised clinical trial</i>. European Journal of Anaesthesiology. 2023;40(9):660-71. doi: 10.1097/eja.0000000000001875. PubMed PMID: WOS:001041449200012.
4. Choi YS, Park KK, Lee B, Nam WS, Kim D-H. Pericapsular Nerve Group (PENG) Block versus Supra-Inguinal Fascia Iliaca Compartment Block for Total Hip Arthroplasty: A Randomized Clinical Trial. Journal of Personalized Medicine. 2022;12(3). doi: 10.3390/jpm12030408. PubMed PMID: WOS:000774863100001.
5. Cui M, Cao Y, Su J, Zhang J. Analgesic efficacy of pericapsular nerve group block in elderly patients with hip replacement under subarachnoid block. Chinese Journal of Anesthesiology. 2020;40(2):199-202. PubMed PMID: CSCD:6743428.
6. Et T, Korkusuz M. Comparison of the pericapsular nerve group block with the intra-articular and quadratus lumborum blocks in primary total hip arthroplasty: a randomized controlled trial. Korean journal of anesthesiology. 2023;76(6):575‐85. doi: 10.4097/kja.23064. PubMed PMID: CN-02630945.
7. Fontes GM, Perez MV, Miashiro EH, Neto AIdS, Grigio TR, Rodrigues LHS. Pericapsular Hip Block Guided by Ultrasonography in Elderly People with Hip Fracture in the Emergency Sector: Clinical Trial. Revista brasileira de ortopedia. 2024;59(2):e284-e96. doi: 10.1055/s-0044-1785494. PubMed PMID: MEDLINE:38606128.
8. Gao X, Zheng Y, Li X, Wang F. Application of pericapsular nerve group block and fascia iliaca compartment block to early analgesia in the elderly patients with hip fracture in emergency department. Chinese Journal of Critical Care Medicine. 2022;42(12):1089-93. PubMed PMID: CSCD:7369294.
9. Kadlimatti DV, Iqbal MS, Kumar S, Harsoor SS, Harshitha K, Awaradi S. Effect of Ultrasound Guided Fascia Iliaca Block and Pericapsular Nerve Group [PENG] For Positioning and Postoperative Analgesia Prior to Spinal Anaesthesia for Hip Surgeries. Journal of cardiovascular disease research. 2024;15(3):997‐1007. doi: 10.48047/jcdr.2024.15.03.115. PubMed PMID: CN-02683243.
10. Keskes M, Ali Mtibaa M, Abid A, Sahnoun N, Ketata S, Derbel R, et al. Pericapsular nerve group block versus fascia iliaca block for perioperative analgesia in hip fracture surgery: a prospective randomized trial. Pan African medical journal. 2023;46:93. doi: 10.11604/pamj.2023.46.93.41117. PubMed PMID: CN-02665712.
11. Lee B, Lee TS, Jang J, Jung HE, Park KK, Choi YS. A Randomized Controlled Trial to Evaluate the Analgesic Effectiveness of Periarticular Injections and Pericapsular Nerve Group Block for Patients Undergoing Total Hip Arthroplasty. Journal of personalized medicine. 2024;14(4). doi: 10.3390/jpm14040377. PubMed PMID: CN-02693204.
12. Li X, Zheng Y, Gao X, Xu H, Liu P, Han Y, et al. Comparison of continuous pericapsular nerve group block and continuous fascia iliaca compartment block for perioperative analgesia in elderly patients undergoing total hip arthroplasty. Journal of Clinical Anesthestology. 2023;39(3):254-9. PubMed PMID: CSCD:7451143.
13. Liang L, Zhang C, Dai W, He K. Comparison between pericapsular nerve group (PENG) block with lateral femoral cutaneous nerve block and supra-inguinal fascia iliaca compartment block (S-FICB) for total hip arthroplasty: a randomized controlled trial. Journal of anesthesia. 2023;37(4):503‐10. doi: 10.1007/s00540-023-03192-6. PubMed PMID: CN-02546111.
14. Lin DY, Morrison C, Brown B, Saies AA, Pawar R, Vermeulen M, et al. Pericapsular nerve group (PENG) block provides improved short-term analgesia compared with the femoral nerve block in hip fracture surgery: a single-center double-blinded randomized comparative trial. Regional anesthesia and pain medicine. 2021;46(5):398‐403. doi: 10.1136/rapm-2020-102315. PubMed PMID: CN-02259079.
15. Liu M, Gao M, Hu Y, Ren X, Li Y, Gao F, et al. Comparison of the Effect of Pericapsular Nerve Group Block Combined with Lateral Femoral Cutaneous Nerve Block and Fascia Iliaca Compartment Block in Patients Undergoing Hip Arthroscopy Under General Anesthesia: a Randomized, Double-Blind Trial. Journal of pain research. 2024;17:1651‐61. doi: 10.2147/jpr.S456880. PubMed PMID: CN-02699146.
16. Malathi K, Jayanthi A, Vasantharani S. COMPARATIVE STUDY OF PERICAPSULAR NERVE GROUP BLOCK VS FEMORAL NERVE BLOCK FOR POSITIONING UNDER SPINAL ANESTHESIA IN SURGERY OF FEMUR FRACTURE UNDER ULTRASOUND GUIDANCE. International journal of academic medicine and pharmacy. 2024;6(1):1693‐6. doi: 10.47009/jamp.2024.6.1.335. PubMed PMID: CN-02678475.
17. Mosaffa F, Taheri M, Manafi Rasi A, Samadpour H, Memary E, Mirkheshti A. Comparison of pericapsular nerve group (PENG) block with fascia iliaca compartment block (FICB) for pain control in hip fractures: a double-blind prospective randomized controlled clinical trial. Orthopaedics & traumatology, surgery & research : OTSR. 2022;108(1):103135. doi: 10.1016/j.otsr.2021.103135. PubMed PMID: CN-02390961.
18. Nuthep L, Klanarong S, Tangwiwat S. The Analgesic effect of adding ultrasound-guided pericapsular nerve group block to suprainguinal fascia iliaca compartment block for hip fracture surgery: a prospective randomized controlled trial. Medicine. 2023;102(44):e35649. doi: 10.1097/md.0000000000035649. PubMed PMID: CN-02620499.
19. Sahoo S, Sahoo NK, Hansda U, Patro SK, Sahu A, Mohanty CR, et al. Ultrasound-guided pericapsular nerve block compared with IV opioids in hip injuries: a randomised controlled trial. American journal of emergency medicine. 2024;81:99‐104. doi: 10.1016/j.ajem.2024.04.016. PubMed PMID: CN-02696642.
20. Scanaliato JP, Christensen D, Polmear MM, Salfiti C, Gaspar PS, Wolff AB. Prospective Single-Blinded Randomized Controlled Trial Comparing Pericapsular Injection Versus Lumbar Plexus Peripheral Nerve Block for Hip Arthroscopy. American journal of sports medicine. 2020;48(11):2740‐6. doi: 10.1177/0363546520943580. PubMed PMID: CN-02158962.
21. Senthil KS, Kumar P, Ramakrishnan L. Comparison of Pericapsular Nerve Group Block versus Fascia Iliaca Compartment Block as Postoperative Pain Management in Hip Fracture Surgeries. Anesthesia, essays and researches. 2021;15(4):352-6. doi: 10.4103/aer.aer_119_21. PubMed PMID: MEDLINE:35422548.
22. Shan T, Han L, Ge D, Wang H, Zhang Y, Bao H. Application of preoperative ultrasound-guided pericapsular nerve group block combined with intraspinal anesthesia for elderly patients in total hip arthroplasty. Journal of Clinical Anesthestology. 2021;37(5):458-61. PubMed PMID: CSCD:6977845.
23. Tang Y, Zhang X, Yi S, Li D, Guo H, Liu Y, et al. Ultrasound-guided pericapsular nerve group (PENG) block for early analgesia in elderly patients with hip fractures: a single-center prospective randomized controlled study. BMC anesthesiology. 2023;23(1):383. doi: 10.1186/s12871-023-02336-1. PubMed PMID: CN-02629524.
24. Vamshi C, Sinha C, Kumar A, Kumar A, Kumari P, Kumar A, et al. Comparison of the efficacy of pericapsular nerve group block (PENG) block versus suprainguinal fascia iliaca block (SFIB) in total hip arthroplasty: a randomized control trial. Indian journal of anaesthesia. 2023;67(4):364‐9. doi: 10.4103/ija.ija_311_22. PubMed PMID: CN-02562385.
25. Wang L, Zhu J, Wang C, Liu Q, Zhang Y. Comparison of analgesic effect of two kinds of nerve block in total hip arthroplasty. Orthopedic Journal of China. 2021;29(21):1995-7. PubMed PMID: CSCD:7084204.
26. Wang QR, Ma T, Hu J, Yang J, Kang PD. Comparison between ultrasound-guided pericapsular nerve group block and anterio quadratus lumborum block for total hip arthroplasty: a double-blind, randomized controlled trial. European review for medical and pharmacological sciences. 2023;27(16):7523‐32. doi: 10.26355/eurrev_202308_33404. PubMed PMID: CN-02594791.
27. Xie Y, Wang M, Li Z, Liu C, Wang Q, Gong W, et al. Ultrasound-guided hip pericapsular nerve group block for pain control in patients with femoral neck fracture during posture changing. Journal of Clinical Anesthestology. 2020;36(8):754-8. PubMed PMID: CSCD:6795946.
28. Ye S, Wang L, Wang Q, Li Q, Alqwbani M, Kang P. Comparison between Ultrasound-Guided Pericapsular Nerve Group Block and Local Infiltration Analgesia for Postoperative Analgesia after Total Hip Arthroplasty: a Prospective Randomized Controlled Trial. Orthopaedic surgery. 2023;15(7):1839‐46. doi: 10.1111/os.13777. PubMed PMID: CN-02578898.
29. Yin H, Zhang W, Shan T, Tan Q, Bao H, Han L, et al. Comparison of efficacy of pericapsular nerve group block combined with lateral femoral cutaneous nerve block versus fascia iliaca compartment block in elderly patients undergoing total hip arthroplasty under general anesthesia. Chinese Journal of Anesthesiology. 2021;41(5):567-70. PubMed PMID: CSCD:7038670.
30. Zheng L, Jo Y, Hwang J, Rhim H, Park E, Oh C, et al. Comparison of the analgesic efficacy of periarticular infiltration and pericapsular nerve group block for total hip arthroplasty: a randomized, non-inferiority study. Annals of palliative medicine. 2022;11(4):1222‐30. doi: 10.21037/apm-21-2785. PubMed PMID: CN-02361069.
31. Zhou M, Xu Q, Zuo D, Wang Z, Zhang M, Liu T, et al. Analgesic effect of pericapsular nerve group block on elderly patients undergoing hip replacement. Vojnosanitetski pregled. 2023;80(8):655‐60. doi: 10.2298/vsp220429002z. PubMed PMID: CN-02624049.

**2.3 Non-randomized control studies (n=18)**

| **No.** | **Author and year** | **Reasons of exclusion** |
| --- | --- | --- |
| 1 | Allard et al., 2021 | Non-randomized control studies |
| 2 | Álvarez et al., 2024 | Non-randomized control studies |
| 3 | Braun et al., 2023 | Non-randomized control studies |
| 4 | Dalal et al., 2023 | Non-randomized control studies |
| 5 | Ellis et al., 2023 | Non-randomized control studies |
| 6 | Fahey et al., 2022 | Non-randomized control studies |
| 7 | Girombelli et al., 2024 | Non-randomized control studies |
| 8 | Khanna et al., 2021 | Non-randomized control studies |
| 9 | Kim et al., 2023 | Non-randomized control studies |
| 10 | Kollmorgen et al., 2022 | Non-randomized control studies |
| 11 | Kukreja et al., 2020 | Non-randomized control studies |
| 12 | Leyba et al., 2024 | Non-randomized control studies |
| 13 | Pai et al., 2024 | Non-randomized control studies |
| 14 | Patel et al., 2022 | Non-randomized control studies |
| 15 | Pires Sousa et al., 2022 | Non-randomized control studies |
| 16 | Sandri et al., 2020 | Non-randomized control studies |
| 17 | Tavares et al., 2022 | Non-randomized control studies |
| 18 | Yusupov et al., 2023 | Non-randomized control studies |

**References**

1. Allard C, Pardo E, de la Jonquière C, Wyniecki A, Soulier A, Faddoul A, et al. Comparison between femoral block and PENG block in femoral neck fractures: A cohort study. PLoS ONE. 2021;16(6 June). doi: 10.1371/journal.pone.0252716.
2. Álvarez LB, López González JM, Miramontes GI, Gómez BMJ, Colon MV, Aguirre AW, et al. Pericapsular Nervous Group Block versus Suprainguinal Fascia Iliaca Block Using the Same Injection Volume in Primary HIP Arthroplasty Prospective Observational Study. International Journal of Clinical Practice. 2024;2024. doi: 10.1155/2024/6952692.
3. Braun AS, Lever JEP, Kalagara H, Piennette PD, Arumugam S, Mabry S, et al. Comparison of Pericapsular Nerve Group (PENG) Block Versus Quadratus Lumborum (QL) Block for Analgesia After Primary Total Hip Arthroplasty Under Spinal Anesthesia: A Retrospective Study. Cureus Journal of Medical Science. 2023;15(12). doi: 10.7759/cureus.50119. PubMed PMID: WOS:001123996400022.
4. Dalal S, John NM, Shelgaonkar VC. A Prospective Observational Study To Determine The Efficacy Of Ultrasound Guided Pericapsular Nerve Group Block For Positional Pain In Hip Fractures. Research Journal of Pharmaceutical, Biological and Chemical Sciences. 2023;14(3):203-8. doi: 10.33887/rjpbcs/2023.14.3.29.
5. Ellis S, Harris JD, Flemming DP, Ellis TJ, Kollmorgen RC. Addition of Pericapsular Nerve Group and Transversus Abdominis Plane Blocks Significantly Reduces Opioid Use in Patients Undergoing Concomitant Hip Arthroscopy and Periacetabular Osteotomy. Cureus. 2023;15(1):e33277. Epub 2023/02/07. doi: 10.7759/cureus.33277. PubMed PMID: 36741622; PubMed Central PMCID: PMCPMC9892016.
6. Fahey A, Cripps E, Ng A, Sweeny A, Snelling PJ. Pericapsular nerve group block for hip fracture is feasible, safe and effective in the emergency department: a prospective observational comparative cohort study. Emergency medicine Australasia. 2022;34(6):884‐91. doi: 10.1111/1742-6723.14013. PubMed PMID: CN-02414922.
7. Girombelli A, Vetrone F, Saglietti F, Galimberti A, Fusaro A, Umbrello M, et al. Pericapsular nerve group block and lateral femoral cutaneous nerve block versus fascia iliaca block for multimodal analgesia after total hip replacement surgery: A retrospective analysis. Saudi J Anaesth. 2024;18(2):218-23. Epub 2024/04/24. doi: 10.4103/sja.sja_881_23. PubMed PMID: 38654885; PubMed Central PMCID: PMCPMC11033891.
8. Khanna S, Prasad K, Sharma V. Pericapsular Nerve Group (PENG) Block for Hip Fracture Surgeries: An Observational Study. Trends in Medical Research. 2021;16(2):37-41. doi: 10.3923/tmr.2021.37.41.
9. Kim DH, Hong G, Lin E, Kim SJ, Beathe J, Wetmore D, et al. Combined Pericapsular Nerve Group Block and Intrapelvic Lateral Femoral Cutaneous Nerve Block Is Associated With Decreased Opioid Consumption After Hip Arthroscopy: A Retrospective Cohort Study. Hss Journal. 2023. doi: 10.1177/15563316231201335. PubMed PMID: WOS:001160748100001.
10. Kollmorgen R, Umerani M, Gollon J, Fleming D, Lewis B, Harris J, et al. Preoperative Pericapsular Nerve Group Block Results in Less Pain, Decreased Narcotic Use, and Quicker Discharge Time Than No Block in Patients Who Were Surgically Treated for Femoroacetabular Impingement Syndrome. Arthroscopy, Sports Medicine, and Rehabilitation. 2022;4(5):e1617-e21. doi: 10.1016/j.asmr.2022.06.004.
11. Kukreja P, Schuster B, Northern T, Sipe S, Naranje S, Kalagara H. Pericapsular Nerve Group (PENG) Block in Combination With the Quadratus Lumborum Block Analgesia for Revision Total Hip Arthroplasty: A Retrospective Case Series. Cureus Journal of Medical Science. 2020;12(12). doi: 10.7759/cureus.12233. PubMed PMID: WOS:000602927100002.
12. Leyba E, Harris H, Gallardo O, Morgan W, Cornelius B. Pericapsular Nerve Group (PENG) Block Results in Significant Opioid Reduction in Total Hip Arthroplasty: A Retrospective Analysis. Journal of perianesthesia nursing : official journal of the American Society of PeriAnesthesia Nurses. 2024;39(2):270-3. doi: 10.1016/j.jopan.2023.08.005.
13. Pai P, Amor D, Lai YH, Echevarria GC. Use and Clinical Relevancy of Pericapsular Nerve Block (PENG) in Total Hip Arthroplasty. Clinical Journal of Pain. 2024;40(5):320-32. doi: 10.1097/ajp.0000000000001196. PubMed PMID: WOS:001201736200002.
14. Patel V, Patel V, Abdallah F, Whelan D, Bansal S, Gabra M, et al. The analgesic benefit of Pericapsular Nerve Group (PENG) block in hip arthroscopic surgery: a retrospective pragmatic analysis at an academic health center. Regional anesthesia and pain medicine. 2022. doi: 10.1136/rapm-2022-103743. PubMed PMID: CN-02459796.
15. Pires Sousa I, Leite da Silva Peixoto CI, Fernandes Coimbra LA, da Costa Rodrigues FM. Comparison of pericapsular nerve group (PENG) block and epidural analgesia following total hip arthroplasty: A retrospective analysis. Rev Esp Anestesiol Reanim (Engl Ed). 2022;69(10):632-9. Epub 2022/11/15. doi: 10.1016/j.redare.2022.10.002. PubMed PMID: 36376187.
16. Sandri M, Blasi A, De Blasi RA. PENG block and LIA as a possible anesthesia technique for total hip arthroplasty. Journal of Anesthesia. 2020;34(3):472-5. doi: 10.1007/s00540-020-02768-w.
17. Tavares BS, Machado RdA, de Arruda UT, de Oliveira LA. DOES PERICAPSULAR ANESTHETIC BLOCK IMPROVE THE POSTOPERATIVE PERIOD IN TRANSTROCHANTERIC FRACTURES? Acta Ortopedica Brasileira. 2022;30(4). doi: 10.1590/1413-785220223004e258190. PubMed PMID: WOS:000886607900010.
18. Yusupov A, Fasulo SM, Castrodad IMD, Kraeutler MJ, Scillia AJ. Improved Pain and Perioperative Outcomes After Hip Arthroscopy With the Pericapsular Nerve Group Block. Arthroscopy-the Journal of Arthroscopic and Related Surgery. 2023;39(2):293-7. doi: 10.1016/j.arthro.2022.08.036. PubMed PMID: WOS:001030908500001.

**2.4 Case Report (n=8)**

| **No.** | **Author and year** | **Reasons of exclusion** |
| --- | --- | --- |
| 1 | Del Buono et al., 2020 | Case Report |
| 2 | Domagalska et al., 2023 | Case Report |
| 3 | Gupta et al., 2021 | Case Report |
| 4 | Marrone et al., 2024 | Case Report |
| 5 | Ng et al., 2022 | Case Report |
| 6 | Niu et al., 2022 | Case Report |
| 7 | Wang et al., 2022 | Case Report |
| 8 | Xu et al., 2022 | Case Report |

**References**

1. Del Buono R, Padua E, Pascarella G, Soare CG, Barbara E. Continuous PENG block for hip fracture: A case series. Regional Anesthesia and Pain Medicine. 2020;45(10):835-8. doi: 10.1136/rapm-2020-101446.
2. Domagalska M, Wieczorowska-Tobis K, Reysner T, Geisler-Wojciechowska A, Grochowicka M, Kowalski G. Pericapsular Nerves Group (PENG) Block in Children under Five Years of Age for Analgesia in Surgery for Hip Dysplasia: Case Report. Journal of Personalized Medicine. 2023;13(3). doi: 10.3390/jpm13030454.
3. Gupta G, Rabbi Q, Bohra V, Shah MM. Protrusio acetabulae as a sequel to septic arthritis of the hip with obturator internus pyomyositis. Journal of Pediatric Orthopaedics Part B. 2021;30(6):572-8. doi: 10.1097/bpb.0000000000000823.
4. Marrone F, Fusco P, Tulgar S, Paventi S, Tomei M, Fabbri F, et al. Combination of Pericapsular Nerve Group (PENG) and Sacral Erector Spinae Plane (S-ESP) Blocks for Hip Fracture Pain and Surgery: A Case Series. Cureus Journal of Medical Science. 2024;16(2). doi: 10.7759/cureus.53815. PubMed PMID: WOS:001174552200010.
5. Ng TK, Lin JA, Sasaki S. A Preliminary Analysis of a Modified Anterior Approach to Hip Pericapsular Neurolysis for Inoperable Hip Fracture Using the IDEAL Framework. Healthcare (Basel). 2022;10(6). Epub 2022/06/25. doi: 10.3390/healthcare10061002. PubMed PMID: 35742053; PubMed Central PMCID: PMCPMC9222877.
6. Niu Z, Xu X, Chu H, Yin J. Anesthetic management of hip fracture in geriatric patient with respiratory and heart failure using pericapsular nerve group block: A case report. Medicine (United States). 2022;101(22):E29478. doi: 10.1097/md.0000000000029478.
7. Wang C-G, Yang Y, Yang M-Y, Wang X-L, Ding Y-L. Analgesic effect of iliopsoas plane block for hip fracture. Perioperative Medicine. 2022;11(1). doi: 10.1186/s13741-022-00254-3. PubMed PMID: WOS:000782418000001.
8. Xu M, He Y, Du B. Pericapsular nerve group block for a child with spina malformation for hip surgery. Journal of Pediatric Surgery Case Reports. 2022;86. doi: 10.1016/j.epsc.2022.102464.

**3. Full-text articles excluded, with reasons (n=9)**

**3.1 Combined with other analgesic techniques (n=7)**

| **No.** | **Author and year** | **Reasons of exclusion** |
| --- | --- | --- |
| 1 | Hu et al., 2023 | Combined with local infiltration analgesia. |
| 2 | Hua et al., 2023 | Combined with patient-controlled intravenous analgesia. |
| 3 | Lin et al., 2022 | Combined with local infiltration analgesia. |
| 5 | Pascarella et al., 2021 | Combined with local infiltration analgesia. |
| 6 | Pascarella et al., 2024 | Combined with lateral femoral cutaneous nerve block. |
| 7 | Yoo et al., 2024 | Combined with lateral femoral cutaneous nerve block. |
| 9 | Zheng et al., 2022 | Combined with intra-articular analgesia. |

**References**

1. Hu J, Wang Q, Hu J, Kang P, Yang J. Efficacy of ultrasound-guided pericapsular nerve group (PENG) block combined with local infiltration analgesia on postoperative pain after total hip arthroplasty: a prospective, double-blind, randomized controlled trial. Journal of arthroplasty. 2023;38(6):1096‐103.
2. Hua H, Zhang D, Wang M, Xu Y, Chen X, Li X, et al. Effect of patient-controlled intravenous analgesia without background infusion of oxycodone combined with pericapsular nerve group block in elderly patients undergoing total hip arthroplasty. Journal of Clinical Anesthestology. 2023;39(5):492-6.
3. Lin DY, Brown B, Morrison C, Fraser NS, Chooi CSL, Cehic MG, et al. The Pericapsular Nerve Group (PENG) block combined with Local Infiltration Analgesia (LIA) compared to placebo and LIA in hip arthroplasty surgery: a multi-center double-blinded randomized-controlled trial. BMC anesthesiology. 2022;22(1):252.
4. Pascarella G, Costa F, Del Buono R, Pulitanò R, Strumia A, Piliego C, et al. Impact of the pericapsular nerve group (PENG) block on postoperative analgesia and functional recovery following total hip arthroplasty: a randomised, observer-masked, controlled trial. Anaesthesia. 2021;76(11):1492‐8.
5. Pascarella G, Costa F, Strumia A, Ruggiero A, Remore LM, Lanteri T, et al. Lateral Femoral Cutaneous Nerve Block or Wound Infiltration Combined with Pericapsular Nerve Group (PENG) Block for Postoperative Analgesia following Total Hip Arthroplasty through Posterior Approach: A Randomized Controlled Trial. Journal of Clinical Medicine. 2024;13(9).
6. Yoo SH, Lee MJ, Beak MH, Kim WJ. Efficacy of Supplemental Ultrasound-Guided Pericapsular Nerve Group (PENG) Block Combined with Lateral Femoral Cutaneous Nerve Block in Patients Receiving Local Infiltration Analgesia after Hip Fracture Surgery: a Prospective Randomized Controlled Trial. Medicina (Kaunas, Lithuania). 2024;60(2).
7. Zheng J, Pan D, Zheng B, Ruan X. Preoperative pericapsular nerve group (PENG) block for total hip arthroplasty: a randomized, placebo-controlled trial. Regional anesthesia and pain medicine. 2022;47(3):155‐60.

**3.2 Other reasons(n=2)**

| **No.** | **Author and year** | **Reasons of exclusion** |
| --- | --- | --- |
| 1 | Mostafa et al., 2023 | The full text is not available. |
| 2 | Zheng et al., 2024 | The full text is not available. |

**References**

1. Mostafa TAH, Mourad MBE, Mohamed NK. Pericapsular nerve group block in hip arthroplasty: a prospective randomized trial. Journal of opioid management. 2023;19(4):313‐20.
2. Zheng J, Feng Z, Zhu J, Kang Y. Application of hip capsule peripheral nerve block in early analgesia in elderly patients with hip fracture. Cirugia y cirujanos. 2024.

**4. Studies eventually included (n=6)**

| **No.** | **Author and year** | **Results** |
| --- | --- | --- |
| 1 | Amato et al., 2022 | The final inclusion. |
| 2 | Chung et al., 2022 | The final inclusion. |
| 3 | Domagalska et al., 2023 | The final inclusion. |
| 4 | Eppel et al., 2023 | The final inclusion. |
| 5 | Kukreja et al., 2023 | The final inclusion. |
| 6 | Lin et al., 2023 | The final inclusion. |

**References**

1. Amato PE, Coleman JR, Dobrzanski TP, Elmer DA, Gwathmey FW, Slee AE, et al. Pericapsular nerve group (PENG) block for hip arthroscopy: a randomized, double-blinded, placebo-controlled trial. Reg Anesth Pain Med. 2022 :rapm-2022-103907.
2. Chung CJ, Eom DW, Lee TY, Park SY. Reduced Opioid Consumption with Pericapsular Nerve Group Block for Hip Surgery: A Randomized, Double-Blind, Placebo-Controlled Trial. Pain Res Manag. 2022;2022:6022380.
3. Domagalska M, Ciftci B, Reysner T, Kolasiński J, Wieczorowska-Tobis K, Kowalski G. Pain Management and Functional Recovery after Pericapsular Nerve Group (PENG) Block for Total Hip Arthroplasty: A Prospective, Randomized, Double-Blinded Clinical Trial. J Clin Med. 2023;12(15):4931.
4. Eppel B, Schneider MM, Gebhardt S, Balcarek P, Sobau C, Wawer Matos J, et al. Pericapsular Nerve Group Block Leads to Small but Consistent Reductions in Pain Between 18 and 24 Hours' Postoperatively in Hip Arthroscopy for Femoroacetabular Impingement Surgery: A Prospective, Randomized Controlled Clinical Trial. Arthroscopy. 2024;40(2):373-380.
5. Kukreja P, Uppal V, Kofskey AM, Feinstein J, Northern T, Davis C, et al. Quality of recovery after pericapsular nerve group (PENG) block for primary total hip arthroplasty under spinal anaesthesia: a randomised controlled observer-blinded trial. Br J Anaesth. 2023;130(6):773-779.
6. Lin X, Liu CW, Goh QY, Sim EY, Chan SKT, Lim ZW, et al. Pericapsular nerve group (PENG) block for early pain management of elderly patients with hip fracture: a single-center double-blind randomized controlled trial. Reg Anesth Pain Med. 2023;48(11):535-539.
