# Supplementary material for: Pericapsular nerve group block reduces opioid use and pain after hip surgery: A systematic review and meta-analysis of randomized controlled trials
Source: PLoS One. 2024 Nov 8;19(11):e0310008. doi: 10.1371/journal.pone.0310008 (PMC11548832; doi:10.1371/journal.pone.0310008)

**Forest plots those were not included in the manuscript**

**Fig 1.** Forest plot for sensitivity analysis of opioid consumption within 24 h after hip surgery.


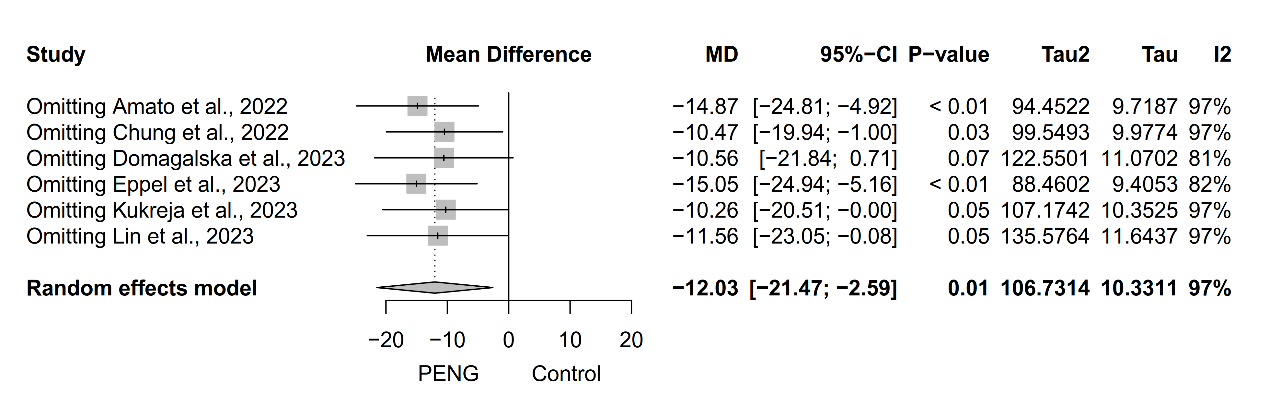


**Fig 2.** Forest plot of the static pain score within 24 h after hip surgery, monitored at 1, 3, 6, and 24 h.


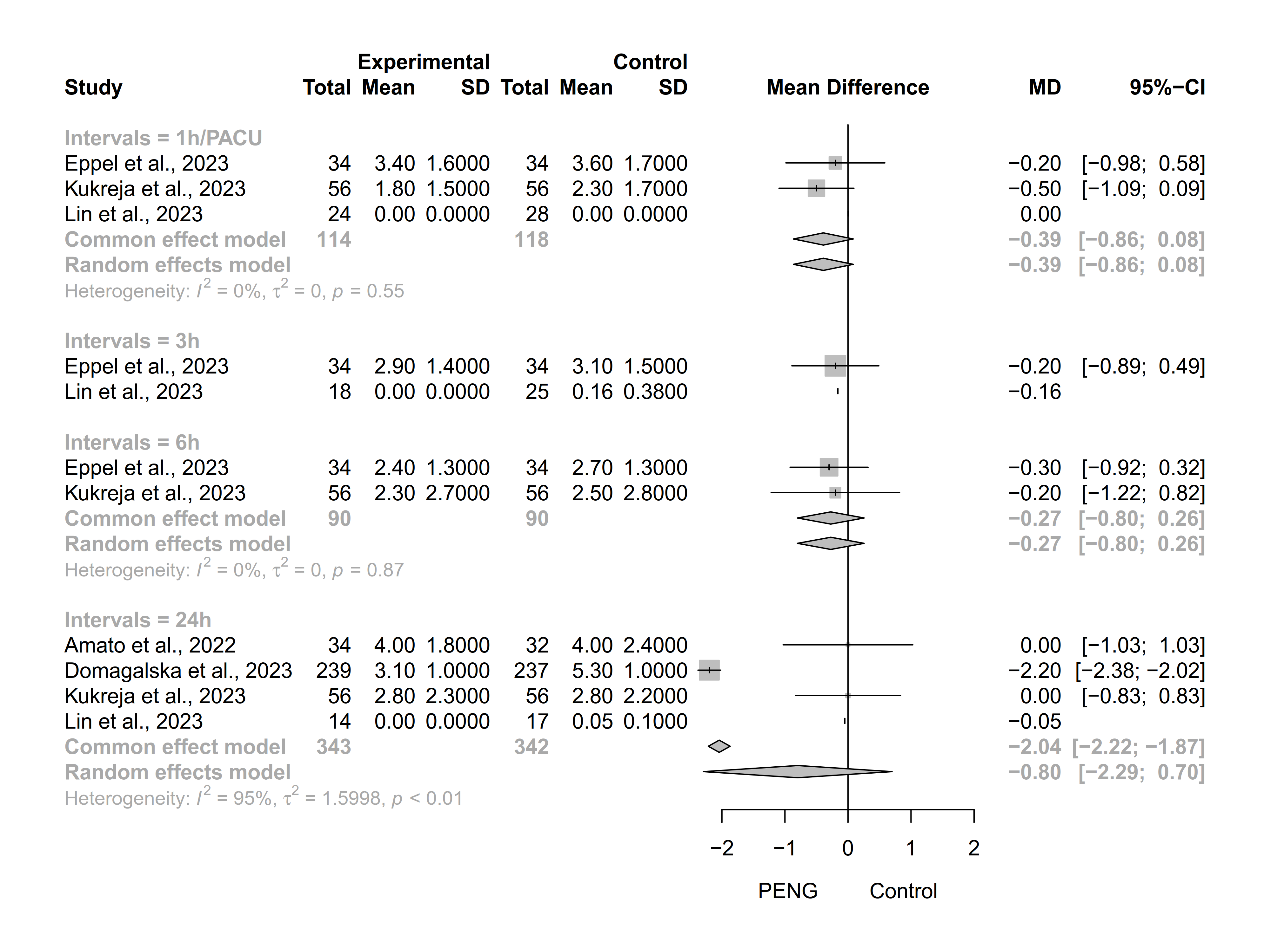


**Fig 3.** Forest plot of sensitivity analysis of static pain scores at 24 h after hip surgery.


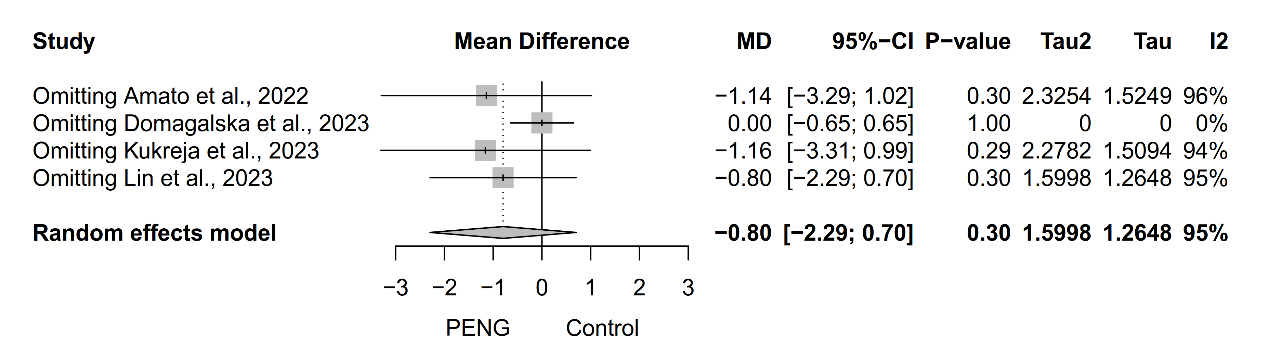


**Fig 4.** Forest plot of the risk of nausea and vomiting.


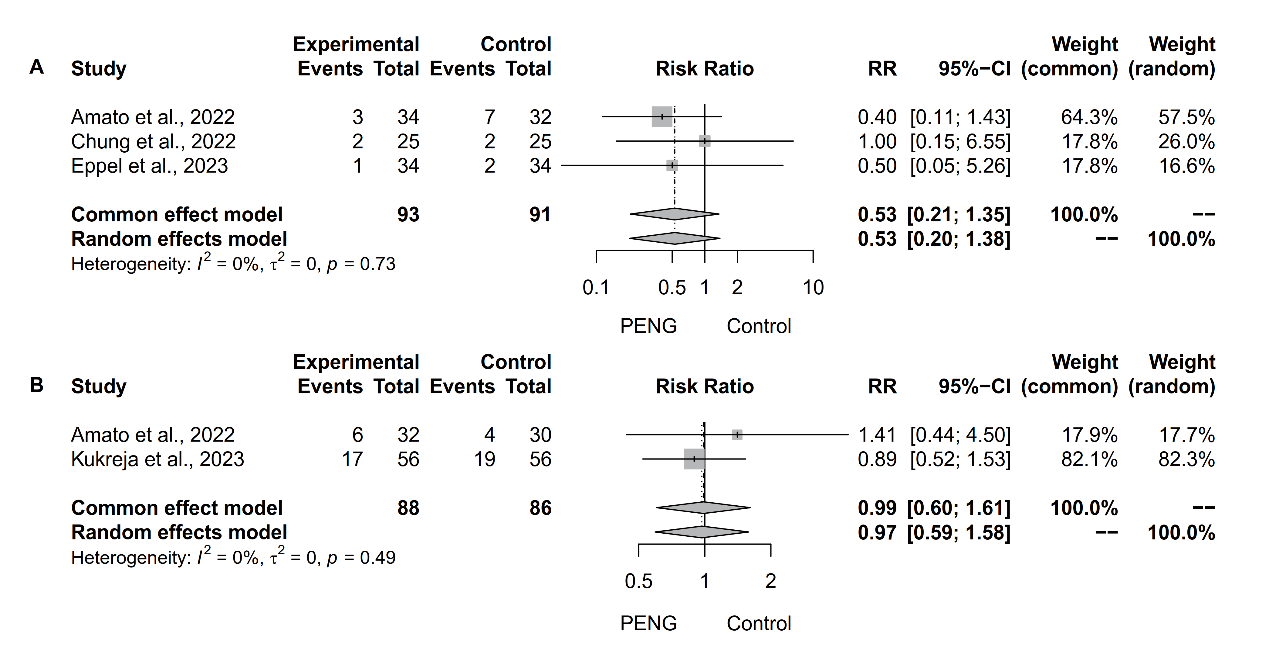


**Fig 5.** Forest plot of patient dissatisfaction.


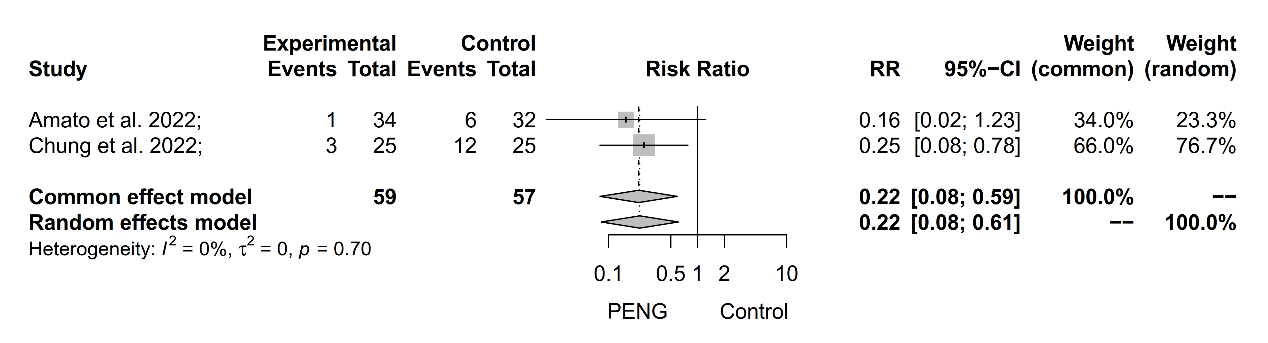

Supplement: S5 Appendix — (DOCX) [file pone.0310008.s005.docx]
